# Early adaptive schemas, emotional regulation, and cognitive flexibility in eating disorders: subtype specific predictors of eating disorder symptoms using hierarchical linear regression

**DOI:** 10.1007/s40519-024-01682-4

**Published:** 2024-08-29

**Authors:** J. S. Mitchell, T. Huckstepp, A. Allen, P. J. Louis, T. E. Anijärv, D. F. Hermens

**Affiliations:** 1 Thompson Institute, University of the Sunshine Coast, Birtinya, QLD Australia; 2 School of Health, University of Sunshine Coast, Sippy Downs, QLD Australia; 3 USCI University, Kuala Lumpur, Malaysia; 4 Clinical Memory Research Unit, Lund University, Lund, Sweden

**Keywords:** Eating disorders, Early adaptive schemas, Cognitive flexibility, Emotional regulation

## Abstract

**Purpose:**

Understanding how early adaptive schemas, cognitive flexibility, and emotional regulation influence eating disorder (ED) symptoms, and whether this differs across diagnostic subtypes is critical to optimising treatment. The current study investigated the relationship between these variables and ED symptomology in individuals self-reporting an ED diagnosis and healthy controls.

**Methods:**

A dataset of 1576 online survey responses yielded subsamples for anorexia nervosa (*n* = 155), bulimia nervosa (*n* = 55), binge eating disorder (*n* = 33), other specified feeding or eating disorder (*n* = 93), and healthy participants (*n* = 505). The hierarchical linear regression analysis included Eating Disorder Examination Questionnaire 6.0 Global Score as the dependent variable; Young Positive Schema Questionnaire, Emotional Regulation Questionnaire, and Cognitive Flexibility Inventory subscale scores as the independent variables; and demographic measures as the covariates.

**Results:**

The number of significant predictors varied considerably by ED sub-group. Amongst the anorexia nervosa, bulimia nervosa, and healthy subsamples, the adaptive schema Self-Compassion and Realistic Expectations was associated with lower ED symptom severity. In comparison, age and body mass index were the strongest predictors for binge eating disorder, whilst the Expressive Suppression (a subscale of the Emotional Regulation Questionnaire) was the strongest predictor for other specified feeding or eating disorders.

**Conclusion:**

Early adaptive schemas, cognitive flexibility, and emotional regulation vary across ED subtype, suggesting the need for tailored treatment that disrupts the self-reinforcing cycle of ED psychopathology. Future research investigating how early adaptive schemas may predict or be associated with treatment response across diagnostic subtypes is needed.

*Level of evidence*: Level IV, evidence obtained from multiple time-series with or without the intervention, such as case studies.

**Supplementary Information:**

The online version contains supplementary material available at 10.1007/s40519-024-01682-4.

## Introduction

### Background

Eating disorders (EDs) comprise both clinically defined and related syndromes characterised by maladaptive eating behaviours, altered body image-related perceptions, and impaired behavioural regulation [1, 2]. Whilst ED subtypes display characteristic symptoms: Anorexia Nervosa (AN), restrictive eating; Bulimia Nervosa (BN), binge eating followed by purging behaviours; Binge Eating Disorder (BED), recurrent episodes of overeating without compensatory behaviours; and Other Specified Feeding or Eating Disorders (OSFED), a range of disordered eating patterns not meeting other diagnostic criteria, there is growing evidence to suggest early maladaptive schemas are a key transdiagnostic risk and maintenance factor across EDs [3].

### Schema therapy and early maladaptive schemas

Accordingly, there is interest in using schema therapy to identify and challenge early maladaptive schemas related to body-image perfectionism for the treatment of EDs [4, 5]. Young [6] defines early maladaptive schemas as themes or patterns consisting of emotions, cognitions, memories, and bodily sensations that distort one’s view of oneself and one’s relationship with others. These patterns are often rigid and pervasive during one’s lifetime, comparable to personality disorder features [7]. Early maladaptive schemas develop when early caregivers do not adequately meet a child’s core emotional needs [6]. This often includes trauma and mistreatment [8], but can also include overprotection from parents [9]. Other contributing factors to early maladaptive schemas include a child’s temperament, culture, and environment [6]. The specific unmet needs are associated with the type of early maladaptive schemas that may develop [10] which, in turn, is linked with the development of an ED [11]. When an early maladaptive schema is triggered, an individual uses a range of coping mechanisms to reduce the associated emotional pain. For the ED populations, such coping mechanisms include constant self-criticism and holding one’s body image to a high standard at the expense of one’s mental well-being [12].

### Early adaptive schemas

In contrast to early maladaptive schemas, early adaptive schemas have been theorised to develop when a child’s core emotional needs are met adequately early in life by primary caregivers [13, 14]. Early maladaptive schemas may influence the development and maintenance of EDs with the number, and content of early maladaptive schemas shown to be associated with disordered eating behaviours and their severity [11, 15]. Crucially, schema therapy treatment includes not just confronting the rigid early maladaptive schemas, but also helping to increase the strengths of early adaptive schemas. These include a broad range of adaptive capacities including that of being compassionate towards and accepting of oneself. Consistent with this, schema therapy treatment has produced positive responses even in severe ED presentations [4, 5]. However, additional research is required to investigate the link between early adaptive schemas and ED symptoms, and whether this varies across ED subtypes as this may inform treatment approach.

In one recent study investigating the relationship between early adaptive schemas and the Eating Disorder Examination Questionnaire 6.0 (EDE-Q) subscales [15], healthy boundaries, emotional openness, and spontaneity, social belonging, and self-care were identified as ‘protective’ against ED symptoms, whilst increased optimism predicted greater ED pathology. Providing an invaluable preliminary investigation, the studied sample (*N* = 388) was clinically heterogenous (50% reported current ED diagnosis). However, part (semi-partial) correlations were not reported, and correlations between predictors in the model were high (*r* > 0.7). Thus, there is a need to (1) explore the associations between early adaptive schemas and ED symptoms across (self-reported) clinical ED subtypes, and (2) apply more stringent multicollinearity thresholds; as regression coefficients and standard errors estimates can be biased even at conventionally acceptable thresholds (i.e. variance inflation factor = 5 or Pearson correlation coefficient = 0.7; [16, 17]. Moreover, the regression models in this study accounted for limited (16–31%) variance in ED symptoms, warranting further investigation of new models incorporating relevant variables to more accurately approximate variability in ED symptoms.

### Emotion regulation

Emotion regulation is defined by Gross [18] as the process which individuals regulate and express their emotions. The two broadest categories of emotion regulation are cognitive reappraisal and expressive suppression. Cognitive appraisal is the process of modifying the meaning of a situation to change the emotional impact, whilst expressive suppression involves inhibiting emotion-expressive behaviour [18, 19]. Individuals with EDs experience higher levels of emotional intensity, have less awareness of their emotions, and use less adaptive emotion regulation strategies compared with healthy populations [20–22], and clinical populations with other non-ED mental health diagnoses [23]. Furthermore, greater emotion dysregulation in individuals with EDs is also associated with more severe clinical features, such as repetitive negative thinking, dysfunctional metacognitions, and greater ED symptomatology [21, 24].

There is some evidence to indicate emotional dysregulation profiles vary by ED subtype. For instance, Mallorquí-Bagué et al. [22] found that individuals with BN, BED, and OSFED exhibit poorer emotional regulation than those with AN. Using an ecological momentary assessment to measure daily emotion dynamics, Williams-Kerver et al. [24] found individuals with AN and BN experienced more intense negative affect than those with BED, whilst, the BN and BED groups showed greater daily emotional fluctuation compared to the AN group. Furthermore, they identified that individuals with BN demonstrated a better ability to differentiate emotions, whilst the AN group had the lowest ability to do so. However, two recent meta-analyses indicate emotion regulation difficulties, particularly awareness, acceptance, rumination and reappraisal, are comparable across the spectrum of EDs, and that their role in EDs may be more nuanced and require a consideration of mediating and moderating variables [25, 26].

Emerging evidence suggests the relationship between early maladaptive schemas and disordered eating may be mediated through emotion dysregulation [27]. For example, in a sample of young Lebanese adults, Gerges and colleagues showed that only one schema domain, namely Disconnection and Rejection (consisting of the negative schemas: Defectiveness, Emotional Deprivation, Emotional Inhibition, Social Isolation, Negative Pessimism and Mistrust/Abuse), had a direct effect on disordered eating behaviour, whilst the other four schema domains (i.e. the impaired autonomy/performance, impaired limits, other-directedness, and over vigilance/inhibition schema) had a significant effect only when mediated through emotional dysregulation.

### Cognitive flexibility

Cognitive flexibility is the ability to modify thinking strategies and behaviour in response to changing environmental stimuli. Dennis and Vander Wal [28] emphasise two distinct aspects of cognitive flexibility that contribute to adaptive thinking as measured by the two subscales of the Cognitive Flexibility Inventory. The first is the tendency to perceive problems as controllable (i.e. Control) and the second is the ability to generate multiple alternative explanations and solutions to difficult situations (i.e. Alternatives). Across ED subtypes there is evidence for impaired cognitive flexibility compared to their healthy counterparts [29, 30], being less able to efficiently switch between tasks. There is limited and conflicting evidence that cognitive inflexibility more pronounced in certain subtypes, with evidence for greater difficulties in AN [31], and BED [32]. Of note, there is evidence that cognitive flexibility may constitute an endophenotype of AN, with no significant difference in cognitive flexibility between individuals in the acute phase of AN and those in the recovered stage [30, 33].

### Aim and hypotheses

Taken together, this evidence highlights the converging (and seemingly inter-related) influence that early adaptive schemas, emotion regulation, and cognitive flexibility has in EDs. However, the degree to which these factors predict ED symptom severity and whether this varies between ED subtypes has yet to be determined. As such, the aim of the current study was to investigate the variability in ED symptomology accounted for by early adaptive schemas, cognitive flexibility, and emotional regulation, in individuals whom self-reported a current clinical diagnosis of AN, BN, BED or OSFED. Understanding whether the contributions of these factors differ across diagnostic subtypes may have implications for how schema therapy is utilised. The current study adhered to a nine-factor structure of the Young Positive Schema Questionnaire as this was previously validated with the current sample [1].

As per the pre-registered protocol (see “Protocol Registration”), it was predicted the inclusion of the early adaptive schemas would account for the most significant proportion of variance in ED symptoms. Specifically, congruent with previous findings [15], we predicted the early adaptive schema’s Self-Compassion and Realistic Expectations, Healthy Self-Control, Emotional Openness and Spontaneity, and Social Belonging would each independently predict lower eating disorder symptomatology across ED subtypes (i.e. act as protective factors).

## Methods

### Protocol registration

The analysis and reporting protocol for this manuscript was registered on the Open Science Framework (OSF; https://doi.org/10.17605/OSF.IO/7SDUZ). Post hoc changes to the statistical analyses include: (1) inclusion of all predictor variables in the hierarchical linear regression model regardless of whether they were significantly correlated with the criterion, (2) univariate outliers were removed on a variable-by-variable basis, and (3) the Shapiro–Wilk test was used instead of Kolmogorov–Smirnov test for the normality of residuals assessment.

### Participants

### Instruments

#### Demographic measures

Age, weight, and height were reported by manual entry, whilst education, nationality, and sex were selected from a list (manual entry required for ‘other’). Body-mass-index (BMI) values were calculated using reported weight and height values.

#### Diagnosis

Participants self-reported if they had a current mental health diagnosis and/or ED diagnosis from a health professional (type not specified) and to indicate the specific diagnosis (i.e. AN, BN, BED, or OSFED).

#### Questionnaire measures

Young Positive Schema Questionnaire: early adaptive schemas were measured using a reduced item (48-items) and factor structure (9 factors) version of the Young Positive Schema Questionnaire [13, 34]. The original 56-item questionnaire has strong reliability (*α* = 0.72–0.89) and validity in psychiatric samples [35]. The reliability and validity of the reduced item and factor version used here has previously been shown with this sample [1], with internal consistency matching previous reports (*α* = 0.77 and 0.92).

Eating Disorder Examination Questionnaire 6.0 (EDE-Q): The EDE-Q Global Score (average of subscales) was used to measure ED symptomatology [36], with higher scores indicating greater ED symptom severity. The EDE-Q Global Score has good internal consistency (*α* = 0.90; [37].

Emotional Regulation Questionnaire (ERQ): Cognitive Reappraisal and Expressive Suppression scores were determined by the ERQ [19]. Total scores are calculated by adding relevant subscale items, with higher scores representative of coping strategy use. The reliability of the two subscales in ED samples has previously been established (Cognitive Reappraisal: *α* = 0.87; Expressive Suppression: *α* = 0.80; [38].

Cognitive Flexibility Inventory (CFI): Cognitive flexibility scores were measured using the CFI [28], with two subscales, Alternatives and Control, calculated. The CFI subscales have demonstrated good internal consistency (Alternatives: *α* = 0.91; Control: *α* = 0.85[28].

### Procedure

Individuals aged ≥ 18 years were recruited through social networking sites (e.g. Facebook) and online forums (e.g. Reddit) via the distribution of a survey using the Qualtrics platform between September 2020 and May 2021. Advertisement of the survey was targeted at ED-related pages and forums to increase the chance of capturing individuals with ED symptoms. The survey was completed online, requiring approximately 25 min. A detailed information sheet including a description of the study and its aims was presented on the first page of the electronic survey. Individuals were informed that their data would be recorded anonymously and participation in the research was voluntary, and they could exit the survey at any time. Consent was obtained via participants selecting a checkbox prior to beginning the survey. After the survey, participants were provided with contact details for support services, and they were also given the opportunity to enter a draw to win one of four $50 AUD Amazon vouchers which were drawn at the completion of the project. The study was approved by the University of the Sunshine Coast Ethics Committee (S201469).

### Statistical approach

The nature of missing data was evaluated using Little MCAR’s test in Statistical Package for the Social Sciences (SPSS), with missing values subsequently imputed using the *miceforest* package [39]. Data formatting, scale coding, univariate outlier detection, and descriptive analysis was performed in Python using the *Pandas *[40]*, Numpy *[41]*, MatPlotLib, Seaborn *[42], *Pinqouin* [43], *Statannotations* [44]*,* and *SciKit* [45] packages, with multivariate outlier detection conducted in R studio using previously published code [46]. Univariate outliers were determined using the median absolute deviation procedure (median ± 2.5 (median absolute deviation * 1.4826); [47]), and multivariate outliers were identified using the Mahalanobis Distance variant—Minimum Covariance Distance (see [46]). Descriptive statistics were tabulated for continuous and categorical variables. Correlations were quantified using a Pearson’s correlation coefficient and presented in the Supplementary Materials.

Inferential statistics (and associated assumption checks) were conducted using the *Pingouin* [43], and *HLR* [48] packages in Python. *HLR* package outputs reported in text were verified against Statistical Package for the Social Sciences outputs to contribute to the package’s validation (see Supplementary Materials for example analysis). Group differences in continuous demographic variables were assessed with a one-way Welch ANOVA to account for unequal variances and sample sizes, with Games–Howell test post hoc comparisons (Games–Howell corrected *p* values).

The hierarchical linear regression analysis included EDE-Q Global Score as the dependent (or criterion) variable; Young Positive Schema Questionnaire, ERQ, and CFI subscale scores as the independent (or predictor) variables; and age, body mass index, and sex as the covariates. Each of the covariates were entered in model one, cognitive flexibility (Alternatives and Control subscales) and emotional regulation (Cognitive Reappraisal and Expressive Suppression subscales) entered in model two, and the nine-factor structure of the Young Positive Schema Questionnaire (Emotional Fulfilment and Stable Attachment, Success, Empathic Consideration, Optimism, Emotional Openness, Self-Compassion and Realistic Expectations, Developed Self, Social Belonging, and Healthy Self-Control) entered in model three. *P* values (< 0.05, uncorrected) and confidence intervals (95%) were reported, with confidence intervals interpreted as per recent discussions (see [49, 50]); confidence intervals represent the probability the range around the point-estimate contains the ‘true’ population effect estimate with repeated sampling (i.e. in the long run), not the range of values with 95% probability (this interpretation is reserved for Bayesian credible intervals).

Post hoc power analysis (see Supplementary Materials G) was conducted using G-power (v.3.1) with significance level *α* = 0.05 as recommended for the use in the clinic research [51].

## Results

### Missing data, dataset splitting and outlier removal

Of the submitted responses (*n* = 1576), 175 participants (11.1%) had some missing data. At a questionnaire level, 0.8–5.6% of responses were missing across EDE-Q questions, 9.5% for ERQ questions, and 7.4% for CFI questions. Missing values were imputed using Multiple Imputation by Chained Equations (MICE; [52]), as there was greater than 5% of participants missing data, which was not missing completely at random (Little MCARs X^2^ [1762] = 2084.73, *p* < 0.001).

After missing value imputation, the complete dataset (*n* = 1576) was split according to self-reported ED diagnosis subtype, resulting in datasets for AN (*n* = 174), BN (*n* = 76), BED (*n* = 47), OSFED (*n* = 108). Participants who reported more than one ED diagnosis subtype (*n* = 100) were not included in the analysis. For participants who did not report a current ED diagnosis (*n* = 1063), a subsample reported a previous ED diagnosis (*n* = 190), and another reported a current mental health diagnosis with no previous ED diagnosis (*n* = 315). The remaining participants were deemed the healthy control sample (*n* = 558).

Across samples, univariate outliers for age and questionnaire scales were present; however, these values were retained as they were within the range of plausible values. Univariate outliers detected for BMI (being > 2.5 * median absolute deviations above the median per sub-group) were removed listwise from the AN (*n* = 11), BN (*n* = 4), BED (*n* = 3), OSFED (*n* = 6), and healthy (*n* = 31) samples. Similarly, multivariate outliers across samples (AN [*n* = 5], BN [*n* = 15], BED [*n* = 11], OSFED [*n* = 7], and healthy [*n* = 15]) were removed listwise. Finally, participants who did not disclose their sex (AN [*n* = 3], BN [*n* = 2], OSFED [*n* = 2], and healthy [*n* = 7]) were removed listwise as sex was included as a model covariate (i.e. not disclosed is uninterpretable). This resulted in reduced final samples for AN (*n* = 155), BN (*n* = 55), BED (*n* = 33), OSFED (*n* = 93), and healthy (*n* = 505).

### Assumptions checks

Using a combination of statistical and visual checks, the assumptions of homoscedasticity and normality of model residuals were largely satisfied, with QQ-plots showing minor deviations from normality in the tails only. Whilst not all predictors were significantly correlated with the dependent variable, all displayed a linear relationship, hence, all predictors were retained in the model. Independence of residuals was only met for the BN dataset, with positive autocorrelation of residuals (Durbin–Watson < 1.5) present in the AN, OSFED and healthy datasets, and negative autocorrelation (Durbin–Watson > 2.5) in the BED dataset. Whilst the data were not of a time-series, we suspected autocorrelation could represent spatial autocorrelation due to hierarchical structuring of the data (i.e. clustering). To verify this, we shuffled the data (by row (i.e. subject)) and recalculated the Durbin–Watson statistics. In each case, the independence of residuals was met (see Supplementary Materials), suggesting some spatial clustering in responses. As the autocorrelation did not change the model and effect estimates, we utilised the unshuffled data, noting the autocorrelation as a limitation of the data collection process.

Across the AN, BN, OSFED, and healthy datasets, multicollinearity did exceed conventional thresholds set out in the protocol [16, 17, 53], and therefore, all variables were retained in the model. Severe multicollinearity was detected in the BED dataset. Consequently, those scales with the highest variance inflation factor values (i.e. Young Positive Schema Questionnaire; Openness, Emotional Fulfilment and Stable Attachment, Success, and Social Belonging) were removed from the model.

### Preliminary analysis

#### Descriptive statistics

A summary of sample demographics is provided in Table 1, with group comparisons for continuous variables. The mean and standard deviations for each Young Positive Schema Questionnaire, ERQ, and CFI subscale are provided in the supplementary materials (Table E1. Across subtypes, mean age varied from 25 to 30 years old, with females accounting for greater than 80% of participants (range = 81.82–95.48%). Post hoc comparisons (see supplementary materials table E2-3) found the healthy sample was significantly older than the AN, BN, and OSFED, but not BED sample. BMI varied from 18.5 to 27, with the AN sample reporting the lowest BMI (*M* = 18.56, *SD* = 2.69), which was significantly lower than all other groups. In contrast, the BED sample reported the highest BMI (*M* = 27.03, *SD* = 5.61), which was a significantly greater than all other groups. We note the elevated AN BMI is suggestive of a heterogenous sample including more mild-to-moderate cases and/or atypical presentations.

**Table 1:** Continuous and categorical descriptive statistics for each eating disorder subtype

	Anorexia nervosa	Bulimia nervosa	Binge eating disorder	OSFED	Healthy	Comparisons
Mean (SD/range)						
Age	25.79 (7.06/18–56)	25.56 (5.22/18–41)	30.55 (11.2/18–54)	25.54 (5.21/18–39)	29.59 (12.38/18–78)	AN < healthy *** BN < healthy *** OSFED < healthy ***
BMI	18.56 (2.69/13.13—26.3)	23.4 (4.61/16.3–34.16)	27.03 (5.61/18.01–38.78)	23.54 (4.93/15.83–39.01)	23.51 (4.89/13.18–38.87)	AN < BED*** AN < BN*** AN < healthy *** AN < OSFED *** BN < BED * Healthy < BED * OSFED < BED *
Count						
Sex						
Male	7 (4.51)	4 (7.27)	6 (18.18)	7 (7.53)	51 (10.1)	
Female	148 (95.48)	51 (92.73)	27 (81.82)	86 (92.47)	454 (89.9)	
Education						
< Grade 10	0 (0.0)	0 (0.0)	2 (6.06)	0 (0.0)	1 (0.2)	
Grade 10	3 (1.94)	1 (1.82)	0 (0.0)	7 (7.53)	21 (4.16)	
Grade 12	48 (30.97)	11 (20.0)	8 (24.24)	30 (32.26)	123 (24.36)	
VET	20 (12.9)	11 (20.0)	8 (24.24)	15 (16.13)	81 (16.04)	
Bachelor’s	62 (40.0)	19 (34.55)	12 (36.36)	28 (30.11)	201 (39.8)	
Masters	20 (12.9)	10 (18.18)	1 (3.03)	12 (12.9)	70 (13.86)	
Phd	2 (1.29)	3 (5.45)	2 (6.06)	1 (1.08)	8 (1.58)	
Nationality						
Australian	11 (7.1)	4 (7.27)	5 (15.15)	11 (11.83)	166 (32.87)	
New Zealand	5 (3.23)	2 (3.64)	1 (3.03)	0 (0.0)	8 (1.58)	
North American	87 (56.13)	29 (52.73)	19 (57.58)	54 (58.06)	156 (30.89)	
South American	1 (0.65)	2 (3.64)	1 (3.03)	2 (2.15)	18 (3.56)	
British	19 (12.26)	5 (9.09)	1 (3.03)	9 (9.68)	44 (8.71)	
Chinese	1 (0.65)	0 (0.0)	0 (0.0)	1 (1.08)	5 (0.99)	
Other	23 (14.84)	12 (21.82)	4 (12.12)	11 (11.83)	98 (19.41)	
Canadian	8 (5.16)	1 (1.82)	2 (6.06)	5 (5.38)	10 (1.98)	
Mental health diagnosis						
Major depressive disorder	74 (47.74)	28 (50.91)	15 (45.45)	35 (37.63)	0 (0.00)	
Bipolar disorder	15 (9.68)	4 (7.27)	5 (15.15)	14 (15.05)	0 (0.00)	
Social anxiety disorder	19 (12.26)	8 (14.55)	8 (24.24)	16 (17.2)	0 (0.00)	
Generalised anxiety disorder	79 (50.97)	25 (45.45)	15 (45.45)	43 (46.24)	0 (0.00)	
Panic disorder	16 (10.32)	3 (5.45)	5 (15.15)	7 (7.53)	0 (0.00)	
Post-traumatic stress disorder	40 (25.81)	10 (18.18)	7 (21.21)	33 (35.48)	0 (0.00)	
Substance use disorder	11 (7.1)	6 (10.91)	3 (9.09)	4 (4.3)	0 (0.00)	
Obsessive compulsive disorder	27 (17.42)	6 (10.91)	7 (21.21)	15 (16.13)	0 (0.00)	
Body dysmorphic disorder	37 (23.87)	12 (21.82)	5 (15.15)	14 (15.05)	0 (0.00)	
Borderline personality disorder	18 (11.61)	7 (12.73)	2 (6.06)	12 (12.9)	0 (0.00)	
Attention-deficit hyperactivity disorder	22 (14.19)	10 (18.18)	7 (21.21)	17 (18.28)	0 (0.00)	
Other	11 (7.1)	3 (5.45)	0 (0.0)	12 (12.9)	0 (0.00)	

*p* < 0.05*, *p* < 0.01**, *p* < 0.001***

*OSFED* other specific feeding or eating disorder, *PhD* Philosophy higher degree, *SD* standard deviation, *VET* vocational education training

#### Correlations

Pearson correlations between continuous descriptive statistics and model variables are reported in the supplementary material as a function of ED subtype (Tables B1-5).

### Primary analysis

Here, we report the final model (step 3) output (see Tables 2, 3, 4, 5, and 6 for full output).

**Table 2:** Anorexia nervosa hierarchical multiple regression output

Variable	B	SE B	β	*T-statistic*	*p value*	Unique (semi-partial-squared) variance (%)	R^2^	Adj R^2^	Δ R^2^	F-change (df)
Step 1							0.057	0.038	0.057	3.022 (3,151)*
Constant	5.285 [3.051, 7.52]	1.131		4.673	< 0.001					
Age	0.011 [−0.015, 0.037]	0.013	0.069	0.871	0.385	0.47				
BMI	−0.097 [−0.165, −0.029]	0.034	−0.222	−2.813	0.006	4.94				
Sex	0.29 [-0.591, 1.17]	0.446	0.051	0.650	0.517	0.26				
Step 2							0.156	0.116	0.099	4.317 (4,147)**
Constant	4.974 [2.571, 7.377]	1.216		4.091	< 0.001					
Age	0.021 [−0.005, 0.047]	0.013	0.125	1.581	0.116	1.44				
BMI	−0.077 [−0.144, −0.01]	0.034	−0.176	−2.277	0.024	2.98				
Sex	0.175 [−0.678, 1.029]	0.432	0.031	0.406	0.685	0.09				
Cognitive reappraisal	−0.197 [−0.378, −0.015]	0.092	−0.194	−2.136	0.034	2.62				
Expressive suppression	0.105 [−0.036, 0.246]	0.071	0.117	1.477	0.142	1.25				
Alternate	0.011 [−0.006, 0.028]	0.009	0.11	1.273	0.205	0.93				
Control	−0.026 [−0.051, −0.001]	0.013	−0.188	−2.087	0.039	2.50				
Step 3							0.376	0.304	0.220	5.410 (9,138)***
Constant	4.412 [2.026, 6.798]	1.207		3.656	< 0.001					
Age	0.021 [−0.002, 0.045]	0.012	0.128	1.780	0.077	1.43				
BMI	0.424 [−0.106, 0.021]	0.032	−0.097	−1.316	0.190	0.78				
Sex	−0.042 [−0.365, 1.214]	0.399	0.075	1.062	0.290	0.51				
Cognitive reappraisal	−0.055 [−0.229, 0.12]	0.088	−0.054	−0.621	0.536	0.17				
Expressive suppression	0.104 [−0.067, 0.276]	0.087	0.116	1.203	0.231	0.65				
Alternate	0.008 [−0.009, 0.024]	0.008	0.076	0.890	0.375	0.36				
Control	−0.004 [−0.029, 0.021]	0.013	−0.029	−0.325	0.745	0.06				
YPSQ_EFSA	−0.022 [−0.208, 0.163]	0.094	−0.025	−0.237	0.813	0.03				
YPSQ_S	−0.143 [−0.317, 0.032]	0.088	−0.144	−1.616	0.108	1.18				
YPSQ_EC	0.03 [−0.156, 0.216]	0.094	0.025	0.320	0.749	0.05				
YPSQ_O	−0.158 [−0.347, 0.031]	0.095	−0.151	−1.656	0.100	1.24				
YPSQ_EOS	0.196 [−0.003, 0.395]	0.101	0.199	1.947	0.054	1.71				
YPSQ_SCRE	−0.469 [−0.712, −0.225]	0.123	−0.356	−3.804	< 0.001	6.54				
YPSQ_DS	−0.058 [−0.185, 0.068]	0.064	−0.07	−0.916	0.361	0.38				
YPSQ_SB	−0.112 [−0.299, 0.075]	0.095	−0.114	−1.182	0.239	0.63				
YPSQ_HSC	0.131 [−0.027, 0.288]	0.080	0.149	1.639	0.103	1.22				

*p* < 0.05*, *p* < 0.01**, *p* < 0.001***

*B* unstandardised beta, *β* standardised beta, *DS* developed self, *EC* empathic consideration, *EFSA* emotional fulfilment and stable attachment, *EOS* emotional openness, *HSC* healthy self-control, *O* optimism, *S* success, *SB* social belonging, *SCRE* self-compassion and realistic expectations, *SE* standard error, *YPSQ* Young Positive Schema Questionnaire, *Δ* change in

**Table 3:** Bulimia nervosa hierarchical multiple regression output

Variable	B	SE B	β	T-statistic	*p* value	Unique (semi-partial-squared) variance (%)	R^2^	Adj R^2^	Δ R^2^	F-change (df)
Step 1							0.201	0.154	0.201	4.281 (3,51)**
Constant	2.271 [−0.729, 5.271]	1.494		1.520	0.135					
Age	−0.051 [−0.109, 0.007]	0.029	−0.241	-1.754	0.085	4.82				
BMI	0.041 [−0.022, 0.104]	0.031	0.171	1.302	0.199	2.65				
Sex	1.271 [0.157, 2.385]	0.555	0.302	2.291	0.026	8.22				
Step 2							0.473	0.394	0.271	6.045 (4,47)**
Constant	1.165 [−1.99, 4.32]	1.568		0.743	0.461					
Age	−0.042 [−0.098, 0.013]	0.028	−0.199	−1.525	0.134	2.61				
BMI	0.036 [−0.021, 0.093]	0.028	0.151	1.282	0.206	1.84				
Sex	0.791 [−0.188, 1.77]	0.487	0.188	1.625	0.111	2.96				
Cognitive reappraisal	−0.174 [−0.492, 0.144]	0.158	−0.137	−1.099	0.277	1.36				
Expressive suppression	0.194 [−0.014, 0.401]	0.103	0.22	1.880	0.066	3.97				
Alternate	0.036 [0.008, 0.063]	0.014	0.307	2.620	0.012	7.7				
Control	−0.034 [−0.07, 0.002]	0.018	−0.258	−1.898	0.064	4.04				
Step 3							0.772	0.675	0.299	5.526 (9,38)***
Constant	4.653 [1.822, 7.483]	1.398		3.328	0.002					
Age	−0.004 [−0.053, 0.045]	0.024	-0.018	−0.162	0.872	0.02				
BMI	−0.005 [−0.054, 0.045]	0.024	−0.019	−0.189	0.851	0.02				
Sex	0.49 [−0.286, 1.266]	0.383	0.117	1.279	0.209	0.98				
Cognitive reappraisal	−0.286 [−0.563, -0.009]	0.137	−0.226	−2.087	0.044	2.62				
Expressive suppression	−0.003 [−0.222, 0.215]	0.108	−0.004	−0.031	0.976	0				
Alternate	0.026 [−0.002, 0.054]	0.014	0.223	1.906	0.064	2.18				
Control	−0.001 [−0.034, 0.032]	0.016	−0.007	−0.055	0.957	0				
YPSQ_EFSA	−0.133 [−0.388, 0.121]	0.126	−0.164	−1.063	0.295	0.68				
YPSQ_S	0.231 [0.001, 0.462]	0.114	0.265	2.033	0.049	2.48				
YPSQ_EC	0.05 [−0.271, 0.37]	0.158	0.03	0.314	0.755	0.06				
YPSQ_O	−0.127 [−0.385, 0.13]	0.127	−0.137	−1.000	0.324	0.6				
YPSQ_EOS	−0.228 [−0.453, -0.004]	0.111	−0.248	−2.062	0.046	2.56				
YPSQ_SCRE	−0.848 [−1.245, -0.451]	0.196	−0.6	−4.322	< 0.001	11.23				
YPSQ_DS	0.015 [−0.162, 0.193]	0.088	0.021	0.176	0.861	0.02				
YPSQ_SB	0.179 [−0.055, 0.413]	0.115	0.213	1.549	0.13	1.44				
YPSQ_HSC	−0.048 [−0.235, 0.138]	0.092	−0.057	−0.525	0.603	0.17				

*p* < 0.05*, *p* < 0.01**, *p* < 0.001***

*B* unstandardised beta, *β* standardised beta, *DS* developed self, *EC* empathic consideration, *EFSA* emotional fulfilment and stable attachment, *EOS* emotional openness, *HSC* healthy self-control, *O* optimism, *S* success, *SB* social belonging, *SCRE* self-compassion and realistic expectations, *SE* standard error, *YPSQ* Young Positive Schema Questionnaire, *Δ* change in

**Table 4:** Binge eating disorder hierarchical multiple regression output

Variable	B	SE B	β	T-statistic	*p* value	Unique (semi-partial-squared) variance (%)	R^2^	Adj R^2^	Δ R^2^	F-change (df)
Step 1							0.542	0.494	0.542	11.427 (3,29)***
Constant	0.864 [−2.287, 4.015]	1.541		0.561	0.579					
Age	−0.063 [−0.1, −0.025]	0.018	−0.439	−3.389	0.002	18.15				
BMI	0.161 [0.088, 0.235]	0.036	0.567	4.488	0.000	31.83				
Sex	−0.023 [−1.106, 1.06]	0.530	−0.006	−0.043	0.966	0				
Step 2							0.722	0.644	0.180	4.060 (4,25)*
Constant	0.132 [−3.724, 3.988]	1.872		0.071	0.944					
Age	−0.063 [−0.098, −0.027]	0.017	−0.439	−3.666	0.001	14.93				
BMI	0.11 [0.039, 0.18]	0.034	0.385	3.204	0.004	11.41				
Sex	0.3 [−0.795, 1.395]	0.532	0.074	0.564	0.578	0.35				
Cognitive reappraisal	−0.446 [−0.752, −0.14]	0.148	−0.367	−3.006	0.006	10.04				
Expressive suppression	0.183 [−0.138, 0.504]	0.156	0.163	1.175	0.251	1.53				
Alternate	0.05 [0.014, 0.087]	0.018	0.338	2.832	0.009	8.91				
Control	−0.028 [−0.087, 0.031]	0.029	−0.129	−0.990	0.332	1.09				
Step 3							0.773	0.638	0.051	0.913 (5,20)
Constant	−2.177 [−7.089, 2.734]	2.355		−0.925	0.366					
Age	−0.075 [−0.117, −0.033]	0.020	−0.523	−3.701	0.001	15.49				
BMI	0.161 [0.054, 0.268]	0.051	0.566	3.127	0.005	11.05				
Sex	0.594 [−0.6, 1.788]	0.572	0.146	1.038	0.312	1.22				
Cognitive reappraisal	−0.6 [−1.067, −0.133]	0.224	−0.493	−2.679	0.014	8.11				
Expressive suppression	0.204 [−0.35, 0.757]	0.265	0.181	0.767	0.452	0.67				
Alternate	0.061 [0.016, 0.106]	0.022	0.411	2.822	0.011	9.01				
Control	−0.038 [−0.111, 0.034]	0.035	−0.175	−1.099	0.285	1.37				
YPSQ_EC	−0.154 [−0.666, 0.358]	0.246	−0.094	−0.626	0.538	0.44				
YPSQ_EOS	0.112 [−0.45, 0.675]	0.269	0.082	0.417	0.681	0.2				
YPSQ_SCRE	0.298 [−0.291, 0.887]	0.282	0.263	1.057	0.303	1.26				
YPSQ_DS	−0.159 [−0.504, 0.187]	0.166	−0.146	−0.956	0.351	1.03				
YPSQ_HSC	0.234 [−0.281, 0.749]	0.247	0.175	0.949	0.354	1.02				

*p* < 0.05*, *p* < 0.01**, *p* < 0.001***

*B* unstandardised beta, *β* standardised beta, *DS* developed self, *EC* empathic consideration, *EOS* emotional openness, *HSC* healthy self-control, *SCRE* self-compassion and realistic expectations, *YPSQ* Young Positive Schema Questionnaire, *Δ* change in

**Table 5:** OSFED hierarchical multiple regression output

Variable	B	SE B	β	T-statistic	*p* value	Unique (semi-partial-squared) variance (%)	R^2^	Adj R^2^	Δ R^2^	F-change (df)
Step 1							0.039	0.007	0.039	1.212 (3,89)
Constant	2.18 [−0.574, 4.934]	1.386		1.573	0.119					
Age	0.001 [−0.061, 0.062]	0.031	0.003	0.031	0.976	0				
BMI	0.057 [−0.008, 0.122]	0.033	0.191	1.752	0.083	3.31				
Sex	0.213 [−0.94, 1.365]	0.580	0.038	0.367	0.715	0.15				
Step 2							0.186	0.118	0.146	3.817 (4,85)**
Constant	1.285 [−1.815, 4.384]	1.559		0.824	0.412					
Age	−0.006 [−0.065, 0.053]	0.030	−0.02	−0.187	0.852	0.03				
BMI	0.052 [−0.01, 0.113]	0.031	0.173	1.674	0.098	2.68				
Sex	0.048 [−1.08, 1.177]	0.568	0.009	0.085	0.932	0.01				
Cognitive reappraisal	−0.239 [−0.514, 0.037]	0.138	−0.195	−1.723	0.088	2.85				
Expressive suppression	0.336 [0.119, 0.552]	0.109	0.325	3.078	0.003	9.08				
Alternate	0.014 [−0.012, 0.04]	0.013	0.118	1.056	0.294	1.07				
Control	0 [−0.038, 0.038]	0.019	0	−0.003	0.997	0				
Step 3							0.384	0.254	0.198	2.719 (9,76)**
Constant	2.345 [−1.156, 5.845]	1.758		1.334	0.186					
Age	−0.04 [−0.1, 0.019]	0.030	−0.142	−1.349	0.181	1.48				
BMI	0.066 [0.004, 0.127]	0.031	0.22	2.117	0.038	3.63				
Sex	−0.481 [−1.695, 0.733]	0.610	−0.087	−0.789	0.432	0.5				
Cognitive reappraisal	−0.155 [−0.465, 0.155]	0.156	−0.127	−0.996	0.323	0.8				
Expressive suppression	0.324 [0.035, 0.613]	0.145	0.314	2.235	0.028	4.05				
Alternate	0.001 [−0.025, 0.027]	0.013	0.006	0.054	0.957	0				
Control	0.006 [−0.037, 0.05]	0.022	0.039	0.293	0.771	0.07				
YPSQ_EFSA	−0.164 [−0.568, 0.241]	0.203	−0.132	−0.806	0.423	0.53				
YPSQ_S	−0.075 [−0.38, 0.229]	0.153	−0.064	−0.494	0.623	0.2				
YPSQ_EC	0.102 [−0.196, 0.4]	0.150	0.076	0.683	0.497	0.38				
YPSQ_O	−0.246 [−0.617, 0.126]	0.187	−0.196	−1.315	0.192	1.4				
YPSQ_EOS	0.314 [−0.055, 0.684]	0.186	0.263	1.694	0.094	1.48				
YPSQ_SCRE	−0.328 [−0.711, 0.056]	0.193	−0.258	−1.702	0.093	0.5				
YPSQ_DS	0.264 [0.01, 0.518]	0.127	0.243	2.074	0.041	3.63				
YPSQ_SB	−0.006 [−0.365, 0.352]	0.180	−0.006	−0.034	0.973	0.8				
YPSQ_HSC	0.196 [−0.093, 0.485]	0.145	0.165	1.354	0.180	4.05				

*p* < 0.05*, *p* < 0.01**, *p* < 0.001***

*B* unstandardised beta, *β* standardised beta, *DS* developed self, *EC* empathic consideration, *EFSA* emotional fulfilment and stable attachment, *EOS* emotional openness, *HSC* healthy self-control, *O* optimism, *S* success, *SB* social belonging, *SCRE* self-compassion and realistic expectations, *SE* standard error, *YPSQ* Young Positive Schema Questionnaire, *Δ* change in

**Table 6:** Healthy sample hierarchical multiple regression output

Variable	B	SE B	β	T-statistic	*P* value	Unique (semi-partial-squared) variance (%)	R^2^	Adj R^2^	Δ R^2^	F-change (df)
Step 1							0.145	0.14	0.145	28.239 (3,501)***
Constant	1.712 [0.529, 2.895]	0.602		2.843	0.005					
Age	−0.055 [−0.067, −0.043]	0.006	−0.395	-8.686	< 0.001	12.88				
BMI	0.074 [0.042, 0.105]	0.016	0.209	4.587	< 0.001	3.59				
Sex	0.577 [0.109, 1.045]	0.238	0.101	2.425	0.016	1				
Step 2							0.386	0.378	0.242	48.923 (4,497)***
Constant	1.489 [0.036, 2.942]	0.740		2.013	0.045					
Age	−0.03 [−0.041, −0.019]	0.006	−0.215	−5.266	< 0.001	3.42				
BMI	0.075 [0.048, 0.102]	0.014	0.212	5.472	< 0.001	3.7				
Sex	0.61 [0.204, 1.016]	0.207	0.107	2.952	0.003	1.08				
Cognitive reappraisal	−0.326 [−0.443, −0.209]	0.059	−0.23	−5.493	< 0.001	3.73				
Expressive suppression	0.32 [0.229, 0.412]	0.047	0.261	6.884	< 0.001	5.85				
Alternate	0.014 [0.001, 0.026]	0.006	0.081	2.096	0.037	0.54				
Control	−0.046 [−0.062, −0.03]	0.008	−0.247	−5.580	< 0.001	3.84				
Step 3							0.513	0.497	0.126	14.044 (9,488)***
Constant	2.608 [1.165, 4.05]	0.734		3.552	< 0.001					
Age	−0.019 [−0.03, −0.008]	0.005	−0.135	−3.442	0.001	1.18				
BMI	0.071 [0.046, 0.096]	0.013	0.201	5.600	< 0.001	3.13				
Sex	0.518 [0.149, 0.886]	0.188	0.09	2.756	0.006	0.76				
Cognitive reappraisal	−0.125 [−0.237, −0.012]	0.057	−0.088	−2.169	0.031	0.47				
Expressive suppression	0.185 [0.072, 0.298]	0.057	0.151	3.224	0.001	1.04				
Alternate	0.013 [0.001, 0.025]	0.006	0.078	2.132	0.034	0.45				
Control	−0.019 [−0.035, −0.002]	0.009	−0.1	−2.173	0.030	0.47				
YPSQ_EFSA	−0.284 [−0.414, −0.154]	0.066	−0.213	−4.293	< 0.001	1.84				
YPSQ_S	−0.008 [−0.135, 0.12]	0.065	−0.005	−0.123	0.902	0				
YPSQ_EC	−0.022 [−0.142, 0.099]	0.061	−0.012	−0.352	0.725	0.01				
YPSQ_O	−0.112 [−0.251, 0.027]	0.071	−0.083	−1.580	0.115	0.25				
YPSQ_EOS	0.072 [−0.057, 0.2]	0.065	0.055	1.099	0.273	0.12				
YPSQ_SCRE	−0.437 [−0.574, −0.301]	0.070	−0.325	−6.291	< 0.001	3.95				
YPSQ_DS	0.08 [−0.019, 0.179]	0.050	0.062	1.579	0.115	0.25				
YPSQ_SB	0.044 [−0.069, 0.156]	0.057	0.034	0.760	0.447	1.18				
YPSQ_HSC	−0.006 [−0.117, 0.105]	0.057	−0.004	−0.106	0.916	0.76				

*p* < 0.05*, *p* < 0.01**, *p* < 0.001***

*B* unstandardised beta, *β* standardised beta, *DS* developed self, *EC* empathic consideration, *EFSA* emotional fulfilment and stable attachment, *EOS* emotional openness, *HSC* healthy self-control, *O* optimism, *S* success, *SB* social belonging, *SCRE* self-compassion and realistic expectations, *SE* standard error, *YPSQ* Young Positive Schema Questionnaire, *Δ* change in

#### Anorexia nervosa

The final model including early adaptive schemas explained an additional 20% of the variance in EDE-Q Global Score (Adj. *R*^2^ = 30.4%), *F*-change (9, 138) = 5.410, *p* < 0.001. Of the predictors, only Self-Compassion and Realistic Expectations accounted for a significant proportion of variance (*β* = −0.356, *p* < 0.001), explaining 6.54% of unique variance in EDE-Q Global Score. Specifically, increased Self-Compassion and Realistic Expectations were associated with decreased ED symptomology.

#### Bulimia nervosa

Model three accounted an additional 27% of the variance (Adj. *R*^2^ = 67.5%) in EDE-Q Global Score, *F*-change (9, 38) = 5.526, *p* < 0.001. Analysis of individual scales showed success (β = 0.265, *p* = 0.049) was the only significant positive predictor of Global Score, whilst Cognitive Reappraisal (β =−0.226, *p* = 0.044), Emotional Openness (β =−0.248, *p* = 0.046), and Self-Compassion and Realistic Expectations (β =−0.600, *p* < 0.001) were significant negative predictors. Higher levels the Success schema was associated with greater ED symptomology, whilst stronger Cognitive Reappraisal, Emotional Openness, Self-Compassion and Realistic Expectations were associated with decreased ED symptoms. Amongst these significant predictors, Self-Compassion and Realistic Expectations accounted for the most (11.23%) unique variance in Global Score, followed by Cognitive Reappraisal (2.62%), Emotional Openness (2.56%), and Success (2.48%).

#### Binge eating disorder

The addition of early adaptive schemas in model three did not account for additional variance (Adj. R^2^ = 63.8%) in EDE-Q Global Score, *F*-change (6, 19) = 0.896, *p* = 0.518. Analysis of individual scales showed Alternatives (β = 0.411, *p* = 0.011) and BMI (β = 0.566, *p* = 0.005) remained significant positive predictors of Global Score, whilst Cognitive Reappraisal (β =−0.493, *p* = 0.014), and age (β =−0.523, *p* = 0.001) remained significant negative predictors. Specifically, higher values of BMI and Alternatives scores were associated with greater ED symptomology, whilst increased age and Cognitive Reappraisal predicted decreased ED symptoms. Amongst these significant predictors, age accounted for the most (15.49%) unique variance in global score, followed by BMI (11.05%), Alternatives (9.01%), and Cognitive Reappraisal (8.11%).

#### Other specified feeding or eating disorders

Model three accounted for an additional 19.8% of the variance in EDE-Q Global Score (Adj. *R*^2^ = 25.4%), *F*-change (9, 76) = 2.719, *p* = 0.008. Expressive Suppression (*β* = 0.314 *p* = 0.028) retained its significant positive association, however, the inclusion of early adaptive schemas led to BMI (*β* = 0.220, *p* = 0.038) and Developed Self (*β* = 0.243, *p* = 0.041) being significant positive predictors. Explicitly, increased Expressive Suppression, Developed Self, and BMI were associated with greater ED symptomology. Amongst these significant predictors, Expressive Suppression (4.05) and Healthy Self-control (4.05%) accounted for the most unique variance, followed by BMI (3.63%), and Developed Self (3.63%).

#### Healthy

Model three accounted for an additional 12.6% of the variance in EDE-Q Global Score (Adj. *R*^2^ = 49.7%), *F*-change (9, 488) = 14.044, *p* < 0.001. The addition of the early adaptive schemas decreased the significance level of Expressive Suppression (*β* = 0.151 *p* = 0.001), Control (*β* =−0.100, *p* = 0.030), and Cognitive Reappraisal (*β* = 0.090, *p* = 0.031), although they remained significant predictors, as did age (*β* =−0.135, *p* < 0.001), BMI (*β* = 0.201 *p* < 0.001), sex (*β* = 0.090, *p* = 0.006), and Alternatives (*β* = 0.078, *p* = 0.034). Emotional Fulfilment and Stable Attachment (*β* =−0.213, *p* < 0.001), and Self-Compassion and Realistic Expectations (*β* =−0.325, *p* < 0.001) were the strongest negative predictors, accounting for 1.84 and 3.95% of unique variance in Global Score, respectively. Of the positive predictors, BMI (3.13%) accounted for the most unique variance, followed by Expressive Suppression (1.04%).

## Discussion

### Summary

Overall, these findings indicate the relationship between early adaptive schemas, cognitive flexibility, emotional regulation, and ED symptoms varies considerably across diagnostic subtypes. In line with the hypotheses, the inclusion of early adaptive schemas accounted for the greatest proportion of variance in ED symptoms in all samples, except for BED. In the case of BED, the model including the CFI and ERQ subscales accounted for the most variance. Contrary to predictions, Healthy Self-Control, Emotional Openness and Spontaneity, and Social Belonging did not independently predict decreased ED symptoms across samples. However, Emotional Openness and Spontaneity did significantly predict lower ED symptoms in BN. Lastly, the hypothesised relationship between Self-Compassion and Realistic Expectations and decreased ED symptoms across diagnoses was partially supported, insofar as it was the strongest predictor of decreased ED symptoms in the AN, BN and healthy samples. In contrast, age and BMI were the strongest predictors for BED, whilst Expressive Suppression was the strongest predictor for OSFED.

Notably, the covariates of age, sex, and BMI displayed ED subtype specific relationships. Age was only a significant predictor of ED symptoms in the BED and healthy control groups, and in both cases, increasing age was associated with lower ED symptoms. Sex was only a significant predictor of ED symptoms in the healthy control group, such that females had significantly greater ED symptoms. BMI was a significant predictor of ED symptoms in the BED, OSFED, and healthy control groups, which predicted greater ED symptoms in all cases.

### Strengths and limits

In assimilating these findings within the existing literature, several limitations must be considered. First, the self-reported nature of the data is a key limitation, and hence, pause is justified when considering the degree of bias inherent in the data, and the overall reliability and validity of the inferences drawn from it. Second, the sample sizes for BN, BED, and OSFED were small compared to the AN and healthy samples, which may contribute to imprecise parameter estimates. Further to this point, a significant number (*n* = 100) of survey respondents who reported multiple EDs were not included in the analysis as concurrent ED diagnoses cannot occur. Third, the values reported suggest the sub-groups somewhat departed from a homogenous grouping. For instance, the healthy sample reported a mean EDE-Q global score of 2.91, which, when compared to previous validation studies in community samples (EDE-Q Global Score = 1.4–1.5; [54]), indicates this sample likely included individuals with undiagnosed EDs. Relatedly, the AN sub-groups BMI was elevated (M = 18.56, SD = 2.69), indicating the sample included mild-to-moderate cases and/or atypical presentations.

Considering the survey was primarily posted on ED-related pages and forums, this is to be expected. Although this may speak to the prevalence of subclinical ED symptoms and heterogeneity of presentations in the population, it introduces a substantial confound, and therefore, we exercised (and recommend) caution when interpreting the results. Finally, the use of a 9-factor structure of the Young Positive Schema Questionnaire was used (see [1]), complicating direct comparisons to studies using the previously validated 14-factor structure [13]. Further studies using populations in Australia should test out the 14-factor structure and test its robustness.

All observed whole and local effects had large effect sizes according to Cohen [55] excluding local effects for OSFED and the Healthy group (see Supplementary Materials G for power analysis). The sample sizes used provided post hoc statistical power values above 0.8 at significance level, *α* = 0.05 for almost all models except for OSFED. Therefore, a limitation here was that the sample size for the investigation of OSFED was not large enough and should be considered in future studies. Another limitation was the use of linear approximation even in cases where conditions for this were violated. However, no non-linear terms were included in the models in this study to reduce model complexity given the limited sample sizes. The use of larger sample sizes in future studies will open possibilities for considering non-linear relationships between predictors and outcomes.

Despite these limitations, the current study provides an informative investigation of the variability in ED symptomology accounted for by early adaptive schemas, cognitive flexibility, and emotional regulation across ED subtypes. Furthermore, this study provides a robust and transparent application of the *HLR* package (with available code) in an effort to promote open-access alternatives to conducting this method of statistical analysis.

### What is already known?

Early adaptive schemas [11], emotion dysregulation [24, 25], and cognitive flexibility [30, 33] have each independently been associated with ED symptom presentation and severity. Emerging research has highlighted their inter-dependence [27, 30, 56], however, limited research has evaluated which factors account for the most variance in ED symptoms and whether this differs across diagnostic subtypes. A recent examination of early adaptive schemas and the Eating Disorder Examination Questionnaire 6.0 (EDE-Q) subscales [15] found healthy boundaries, emotional openness, and spontaneity, social belonging, and self-care were ‘protective’ against ED symptoms, whilst increased optimism predicted greater ED pathology. However, the studied sample (*N* = 388) was clinically heterogenous (50% reported current ED diagnosis).

### What does this study add?

The current findings support previous research indicating an association between ED symptom severity and emotion regulation [21, 22], cognitive flexibility [33], and early adaptive schemas [15]. Specifically, our findings are in accordance with existing literature indicating BED, BN, and AN display a similar emotional regulation profile [20, 22], whilst BED displays a unique profile. For example, we found the ERQ (specifically Cognitive Reappraisal) and CFI (specifically Alternatives) accounted for a significant portion of variance in BED symptom severity, whilst early adaptive schemas showed no such association. It is worth noting that Svaldi et al. [23] previously proposed that individuals with BED might possess superior abilities to recognise and regulate emotions compared to other ED subtypes. In addition, individuals with BED have been observed to use both positive and negative metacognitive beliefs regarding binge eating as a coping strategy to reduce worry and unpleasant emotions [57]. In terms of cognitive flexibility, several previous studies have not included BED in comparative analyses with other ED types [29, 58]. However, in support of the present finding, one study found that individuals with BED exhibited poorer cognitive flexibility than those with AN [32].

Relatedly, our findings suggest that early adaptive schemas may serve as maintenance factors for eating psychopathology in AN and BN, but not in BED. This suggests the potential for disorder-specific treatment strategies, individuals with AN and BN may benefit from attachment-based therapies targeting core emotional needs. Consistent with the ‘protective’ role of Healthy Boundaries reported by Maher [15], Self-Compassion and Realistic Expectations was associated with decreased ED symptom severity in AN, BN, and healthy participants. This contributes to the robust evidence base highlighting perfectionism (i.e. Unrelenting Standards, the antithesis of Realistic Expectations) as a key factor in maintaining ED pathology [3]. Notably, several studies have found an association between perfectionism and low self-compassion [59, 60], a particularly important association in the context of EDs. Individuals with EDs who fear self-compassion tend to have worse treatment outcomes [61], which has been previously associated with two factors [62]. First is a fear of losing personal standards, including perceptions of weakness, fear of reduced motivation, and concerns about being seen as failures. Second, is a fear of emotional vulnerability and feeling unworthy of compassion. This fear and self-compassion can be explained by the challenges faced by individuals with histories of trauma, abuse, neglect, or unmet emotional needs in forming attachment relationships. During therapy, efforts to encourage healthy attachments can reactivate their attachment system, triggering grief and unpleasant emotions they have learned to avoid. The re-emergence of these difficult feelings can pose a barrier to recovery.

In terms of treatment implications, schema therapy presents as a promising of option for anorexia nervosa (AN) and bulimia nervosa (BN), as it can progressively challenge high standards and self-criticism whilst promoting self-compassion and realistic expectations. Although research supports schema therapies effectiveness [5], its specific impact on enhancing self-compassion across different ED presentations requires further investigation. For instance, cognitive flexibility and emotion regulation appear to influence binge eating disorder (BED) more than early adaptive schemas, suggesting that therapies like metacognitive therapy [63], and dialectical behavioural therapy [64], respectively. However, whilst there exists some emerging evidence comparing these therapies to cognitive behaviour therapy [65], the comparative effectiveness with respect schema therapy for EDs remains a matter for future research.

## Conclusion

The current study provides preliminary evidence that early adaptive schemas, cognitive flexibility, and emotional regulation are associated with ED symptoms in a subtype specific manner. Future experimental research is needed to better understand the relationship between these factors and EDs symptoms, and the role they may have in guiding schema-focussed therapies.

### Supplementary Information

Below is the link to the electronic supplementary material.

Supplementary material 1.

Supplementary material 2.

## Data Availability

An example of the study analysis code is provided in the supplementary materials. The package used for the primarily analysis can be accessed at https://github.com/teanijarv/HLR, and the code to reproduce the supplementary material example analysis can be found at https://github.com/JulesMitchell/SchemaHLR. The (de-identified) data that support the findings of this study are available from the corresponding author upon request.
