# Supplementary material for: Early adaptive schemas, emotional regulation, and cognitive flexibility in eating disorders: subtype specific predictors of eating disorder symptoms using hierarchical linear regression
Source: Eat Weight Disord. 2024 Aug 29;29(1):54. doi: 10.1007/s40519-024-01682-4 (PMC11362190; doi:10.1007/s40519-024-01682-4)
Supplement: Supplementary file 1 — Supplementary material 1. [file 40519_2024_1682_MOESM1_ESM.docx]

**Supplementary Materials**

**Assumption Checks (A-D)**

1. **Normality of Residuals and Homoscedasticity**

**Table A1.** Normality of residuals and homoscedasticity checks for each sample

| Test | AN | BN | BED | OSFED | Healthy |
| --- | --- | --- | --- | --- | --- |
| Shapiro Wilks p-value | 0.0002 | 0.029 | 0.948 | 0.0002 | 0.302 |
| Shapiro Wilks Passed | No | No | Yes | No | Yes |

Abbreviations: AN; Anorexia Nervosa, BED; binge eating disorder, BN; Bulimia Nervosa, OSFED; other specified feeding or eating disorder.

**
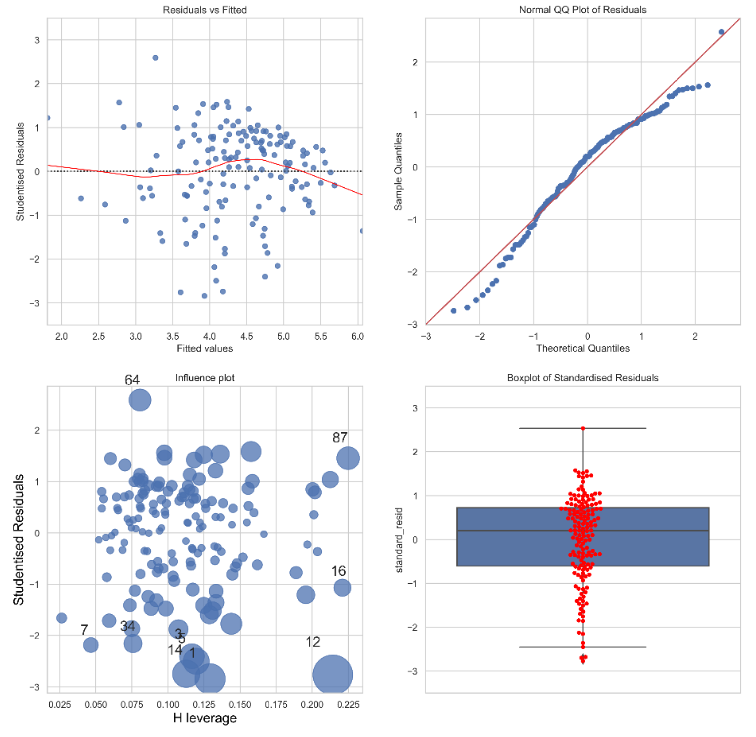
Figure A1.** Anorexia Nervosa


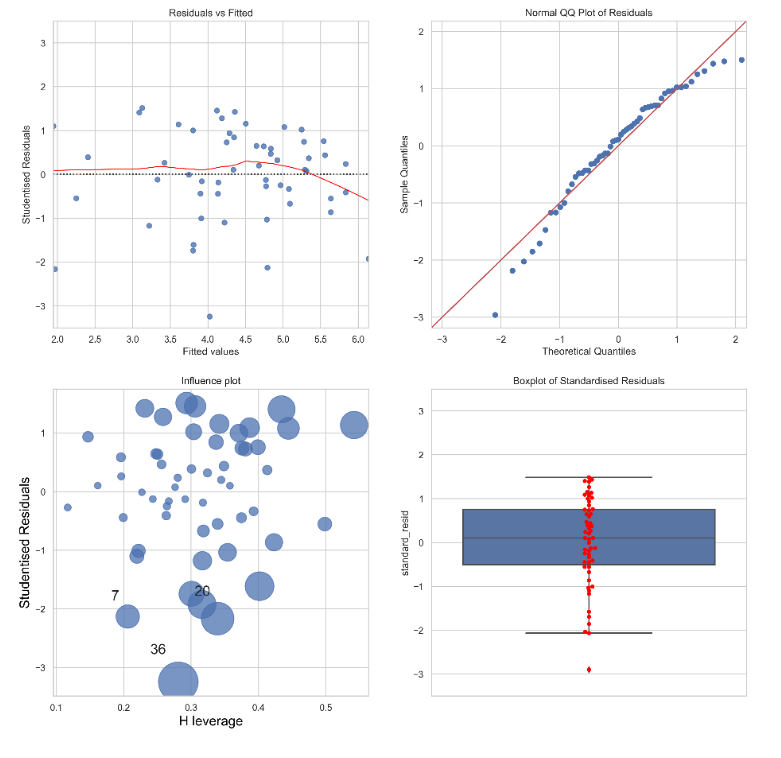
**Figure A2.** Bulimia Nervosa

**
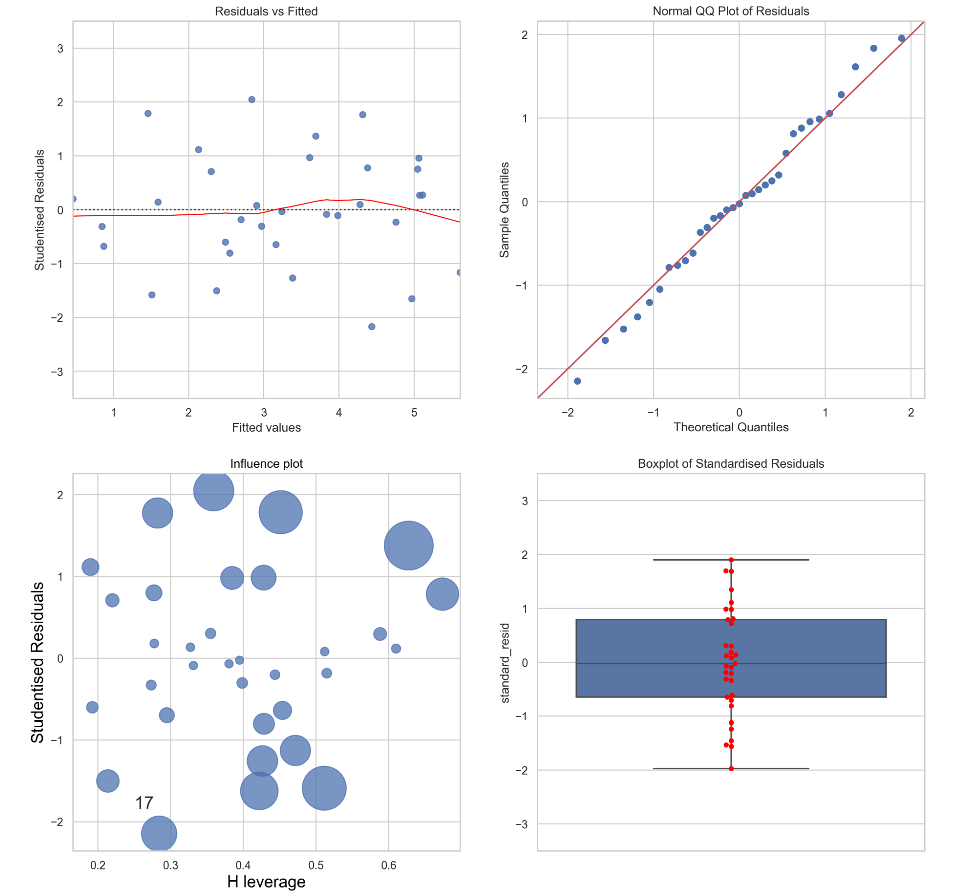
Figure A3.** Binge Eating Disorder

**
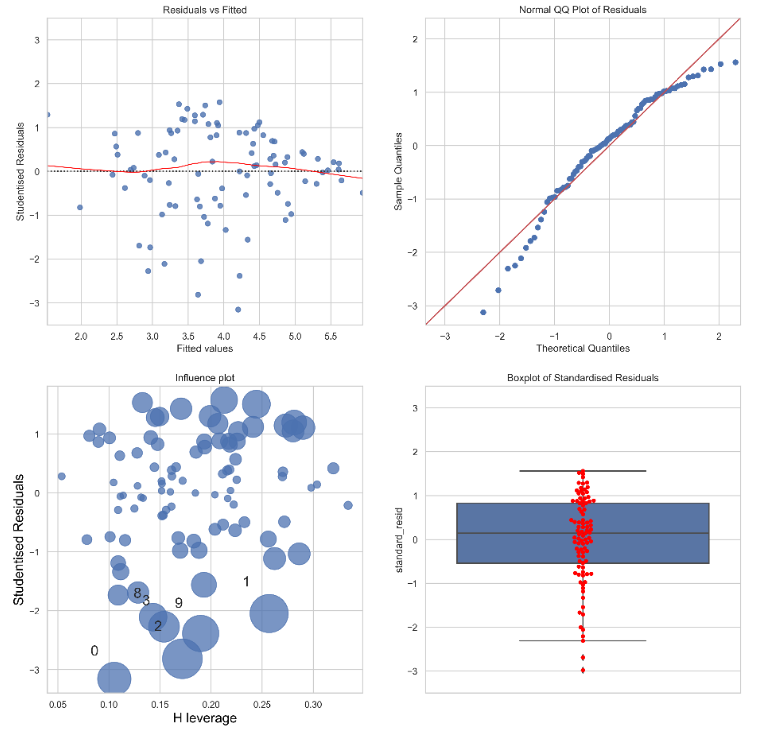
Figure A4.** OSFED

**
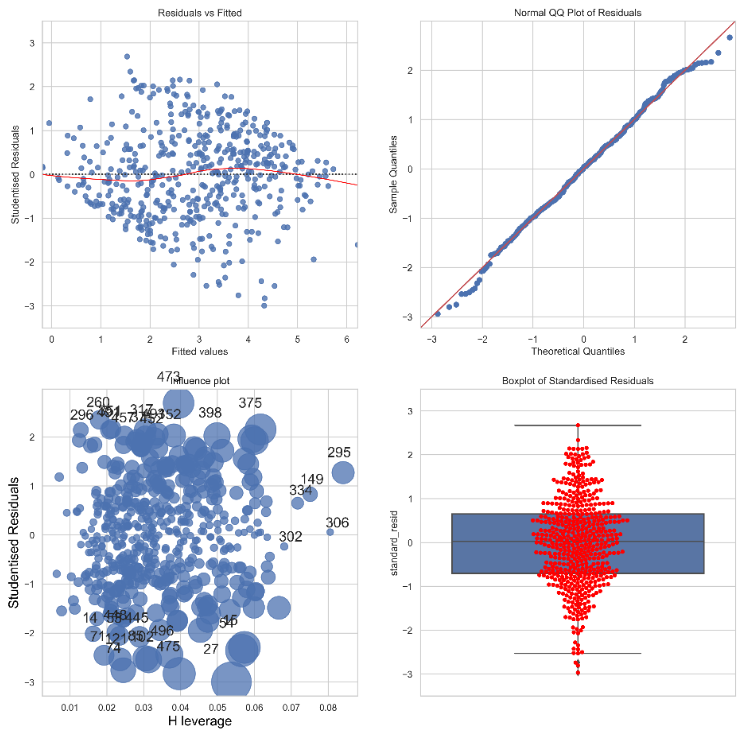
Figure A5.** Healthy

1. **Linearity**

Table B1. Linearity of model predictor checks for each sample

| Variable | Test | AN | BN | BED | OSFED | Healthy |
| --- | --- | --- | --- | --- | --- | --- |
| Age | Pearson r | 0.071 | -0.282 | -0.471 | 0.065 | -0.318 |
|  | p-value | 0.379 | 0.037 | 0.006 | 0.533 | 0 |
| Sex | Pearson r | 0.045 | 0.378 | 0.152 | 0.049 | 0.116 |
|  | p-value | 0.578 | 0.004 | 0.397 | 0.642 | 0.009 |
| BMI | Pearson r | -0.222 | 0.111 | 0.592 | 0.194 | 0.036 |
|  | p-value | 0.005 | 0.42 | 0 | 0.062 | 0.424 |
| CR | Pearson r | -0.211 | -0.249 | -0.429 | -0.2 | -0.403 |
|  | p-value | 0.008 | 0.067 | 0.013 | 0.055 | 0 |
| ES | Pearson r | 0.152 | 0.355 | 0.149 | 0.348 | 0.396 |
|  | p-value | 0.059 | 0.008 | 0.409 | 0.001 | 0 |
| Alt | Pearson r | -0.046 | 0.285 | 0.189 | 0.005 | -0.134 |
|  | p-value | 0.569 | 0.035 | 0.293 | 0.964 | 0.002 |
| Ctrl | Pearson r | -0.28 | -0.499 | -0.238 | -0.186 | -0.459 |
|  | p-value | 0 | 0 | 0.181 | 0.075 | 0 |
| YPSQ_EFSA | Pearson r | -0.345 | -0.535 | N/A | -0.356 | -0.564 |
|  | p-value | 0 | 0 | N/A | 0 | 0 |
| YPSQ_S | Pearson r | -0.262 | -0.2 | N/A | -0.151 | -0.314 |
|  | p-value | 0.001 | 0.144 | N/A | 0.148 | 0 |
| YPSQ_EC | Pearson r | 0.032 | -0.011 | -0.235 | 0.161 | -0.192 |
|  | p-value | 0.69 | 0.938 | 0.188 | 0.123 | 0 |
| YPSQ_O | Pearson r | -0.42 | -0.555 | N/A | -0.338 | -0.556 |
|  | p-value | 0 | 0 | N/A | 0.001 | 0 |
| YPSQ_EOS | Pearson r | -0.131 | -0.44 | -0.348 | -0.219 | -0.372 |
|  | p-value | 0.104 | 0.001 | 0.047 | 0.035 | 0 |
| YPSQ_SCRE | Pearson r | -0.516 | -0.77 | -0.603 | -0.446 | -0.623 |
|  | p-value | 0 | 0 | 0 | 0 | 0 |
| YPSQ_DS | Pearson r | -0.161 | -0.169 | -0.272 | 0.132 | -0.303 |
|  | p-value | 0.045 | 0.218 | 0.125 | 0.208 | 0 |
| YPSQ_SB | Pearson r | -0.297 | -0.393 | N/A | -0.337 | -0.402 |
|  | p-value | 0 | 0.003 | N/A | 0.001 | 0 |
| YPSQ_HSC | Pearson r | -0.038 | -0.139 | -0.354 | 0.005 | -0.342 |
|  | p-value | 0.637 | 0.311 | 0.043 | 0.962 | 0 |
|  | Linearity passed | No: Age, Sex, EmReg_ES, CFI_Alt, YPSQ_EC, YPSQ_EOS, YPSQ_HSC | No: BMI, EmReg_CR, YPSQ_S, YPSQ_EC, YPSQ_DS, YPSQ_HSC | No: | No: Age, Sex, BMI, EmReg_CR, CFI_Alt, CFI_Ctrl, YPSQ_EC, YPSQ_DS, YPSQ_HSC, YPSQ_S | No: BMI |

Abbreviations: Alt; alternate, AN; Anorexia Nervosa, BED; binge eating disorder, BMI; body mass index, BN; Bulimia Nervosa, BED, CR; cognitive reappraisal, Ctrl; control, DS; developed self, EC; empathic consideration, EDE_Q_E; eating disorder questionnaire eating concern, EDE_Q_G; eating disorder questionnaire global score, EDE_Q_R; eating disorder questionnaire restraint, EDE_Q_S; eating disorder questionnaire shape concern, EDE_Q_W; eating disorder questionnaire weight concern, EFSA; emotional fulfilment and stable attachment, EOS; emotional openness, ES; expressive suppression, HSC; healthy self-control, O; openness, OSFED; other specified feeding or eating disorder, S; success, SB; social belonging, SCRE; self-compassion and realistic expectations, YPSQ; young positive schema questionnaire.

1. **Autocorrelation**

Table C1. Autocorrelation checks for each sample

| Test | AN | | BN | | BED | | OSFED | | Healthy | |
| --- | --- | --- | --- | --- | --- | --- | --- | --- | --- | --- |
|  | Unchanged | Shuffled | Unchanged | Shuffled | Unchanged | Shuffled | Unchanged | Shuffled | Unchanged | Shuffled |
| Durbin Watson Statistic | 1.188 | 2.076 | 1.936 | N/A | 2.686 | 1.851 | 1.03 | 1.810 | 1.331 | 1.975 |
| Durbin Watson Passed | No | Yes | Yes | N/A | No | Yes | No | Yes | No | Yes |

Abbreviations: AN; Anorexia Nervosa, BED; Binge Eating Disorder, BN; Bulimia Nervosa, OSFED; Other Specified Feeding or Eating Disorder.

1. **Multicollinearity**

Table D1. Multicollinearity checks for each sample

| Test | AN | BN | BED | OSFED | Healthy |
| --- | --- | --- | --- | --- | --- |
| Pairwise correlations passed (<.07) | Yes | Yes | Yes | No | No |
| VIF passed (< 5) | Yes | Yes | No | Yes | Yes |
| VIF Predictors Failed | N/A | N/A | YPSQ_SCRE = 5.48 | N/A | N/A |

Abbreviations: AN; Anorexia Nervosa, BED; Binge Eating Disorder, BN; Bulimia Nervosa, OSFED; Other Specified Feeding or Eating Disorder, N/A; Not Applicable, SCRE; Self Compassion and Realistic Expectations, YPSQ; Young Positive Schema Questionnaire, VIF; Variation Inflation Factor.

**Preliminary Analyses (E-F)**

1. **Descriptive Statistics**

Table E1. Sample mean and standard deviations for sub-scales of the Eating Disorder Examination Questionnaire, Emotional Regulation Questionnaire, Cognitive Flexibility Inventory, and Young Positive Schema Questionnaire.

|  | AN | BN | BED | OSFED | Healthy |
| --- | --- | --- | --- | --- | --- |
| Mean (SD) | | | | | |
| EDE-Q Global | 4.35 (1.17) | 4.38 (1.1) | 3.27 (1.6) | 3.96 (1.47) | 2.91 (1.73) |
| EDE-Q Restraint | 4.19 (1.43) | 4.19 (1.43) | 2.5 (1.59) | 3.61 (1.85) | 2.8 (1.97) |
| EDE-Q Weight Concern | 4.63 (1.11) | 4.63 (1.11) | 3.42 (1.81) | 4.29 (1.63) | 3.19 (1.85) |
| EDE-Q Shape Concern | 4.7 (1.28) | 4.7 (1.28) | 3.76 (1.83) | 4.33 (1.61) | 3.27 (1.87) |
| EDE-Q Eating Concern | 3.8 (1.35) | 3.8 (1.35) | 3.1 (1.77) | 3.37 (1.47) | 2.16 (1.8) |
| Cognitive Reappraisal | 3.8 (1.16) | 3.8 (0.87) | 4.25 (1.31) | 3.98 (1.21) | 4.47 (1.22) |
| Emotional Suppression | 4.69 (1.31) | 4.5 (1.25) | 4.28 (1.42) | 4.49 (1.43) | 3.9 (1.4) |
| Alternate | 69.15 (11.76) | 69.53 (9.44) | 67.12 (10.77) | 69.11 (12.63) | 71.21 (10.38) |
| Control | 22.21 (8.39) | 23.02 (8.36) | 25.06 (7.27) | 24.23 (9.02) | 29.97 (9.33) |
| YPSQ Emotional Fulfilment and Stable Attachment | 3.05 (1.31) | 2.84 (1.36) | 3.71 (1.22) | 2.98 (1.19) | 3.9 (1.29) |
| YPSQ Success | 3.93 (1.19) | 3.99 (1.26) | 4.19 (1.25) | 3.89 (1.26) | 4.44 (1.13) |
| YPSQ Empathic Consideration | 4.34 (0.98) | 4.3 (0.67) | 4.35 (0.98) | 4.28 (1.1) | 4.42 (0.97) |
| YPSQ Openness | 2.6 (1.13) | 2.54 (1.18) | 3.33 (1.33) | 2.7 (1.18) | 3.61 (1.29) |
| YPSQ Emotional Openness | 3.09 (1.19) | 3.08 (1.2) | 3.85 (1.16) | 3.08 (1.23) | 3.71 (1.32) |
| YPSQ Self-Compassion and Realistic Expectations | 2.18 (0.89) | 2.11 (0.78) | 3.06 (1.41) | 2.5 (1.16) | 3.28 (1.28) |
| YPSQ Developed Self | 4.13 (1.4) | 4.17 (1.47) | 3.98 (1.47) | 4.47 (1.35) | 4.52 (1.35) |
| YPSQ Social Belonging | 2.74 (1.2) | 2.51 (1.31) | 3.48 (1.38) | 2.73 (1.38) | 3.45 (1.34) |
| YPSQ Healthy Self Control | 3.85 (1.34) | 3.23 (1.3) | 3.39 (1.19) | 3.44 (1.24) | 3.88 (1.2) |

Abbreviations: AN; Anorexia Nervosa, BMI; body mass index, BN; Bulimia Nervosa, BED; Binge Eating Disorder, DF; degrees of freedom, EDE-Q; Eating Disorder Examination Questionnaire, OSFED; Other Specified Feeding or Eating Disorder, SD; standard deviation, YPSQ; Young Positive Schema Questionnaire.

Table E2. One-Way Welch ANOVA for Continuous Demographic Variables. Note: Asterisks indicate p-value significance; * < 0.05, ** < 0.01, *** < 0.001.

|  | DF (1) | DF (2) | F-statistic | P-value | Partial eta-squared |
| --- | --- | --- | --- | --- | --- |
| Age | 4 | 151 | 10.12 | *** | 0.03 |
| Height | 4 | 138 | 1.18 | 0.324 | 0.01 |
| Weight | 4 | 138 | 76.3 | *** | 0.16 |
| BMI | 4 | 136 | 80.59 | *** | 0.17 |

Abbreviations: BMI; body mass index, DF; degrees of freedom.

Table E3. Post-Hoc Games-Howell for Continuous Demographic Variables. Note: Asterisks indicate p-value significance; * < 0.05, ** < 0.01, *** < 0.001.

| Variable | A | B | mean(A) | mean(B) | Difference | SE | T- statistic | DF | P-value | Partial eta-square |
| --- | --- | --- | --- | --- | --- | --- | --- | --- | --- | --- |
| Age | AN | BED | 25.79 | 30.55 | -4.76 | 2.03 | -2.34 | 38 | 0.154 | 0.083 |
|  | AN | BN | 25.79 | 25.56 | 0.22 | 0.9 | 0.25 | 128 | 0.999 | 0 |
|  | AN | Healthy | 25.79 | 29.59 | -3.8 | 0.79 | -4.8 | 457 | *** | 0.027 |
|  | AN | OSFED | 25.79 | 25.54 | 0.25 | 0.78 | 0.32 | 236 | 0.998 | 0 |
|  | BED | BN | 30.55 | 25.56 | 4.98 | 2.07 | 2.4 | 40 | 0.135 | 0.089 |
|  | BED | Healthy | 30.55 | 29.59 | 0.96 | 2.03 | 0.47 | 37 | 0.989 | 0.002 |
|  | BED | OSFED | 30.55 | 25.54 | 5.01 | 2.02 | 2.48 | 37 | 0.118 | 0.107 |
|  | BN | Healthy | 25.56 | 29.59 | -4.02 | 0.89 | -4.5 | 135 | *** | 0.028 |
|  | BN | OSFED | 25.56 | 25.54 | 0.03 | 0.89 | 0.03 | 113 | 1 | 0 |
|  | Healthy | OSFED | 29.59 | 25.54 | 4.05 | 0.77 | 5.25 | 320 | *** | 0.03 |
| Height | AN | BED | 1.66 | 1.69 | -0.03 | 0.02 | -1.85 | 40 | 0.358 | 0.043 |
|  | AN | BN | 1.66 | 1.66 | 0 | 0.01 | 0.37 | 94 | 0.996 | 0.001 |
|  | AN | Healthy | 1.66 | 1.67 | -0.01 | 0.01 | -0.98 | 296 | 0.865 | 0.002 |
|  | AN | OSFED | 1.66 | 1.66 | 0 | 0.01 | -0.1 | 175 | 1 | 0 |
|  | BED | BN | 1.69 | 1.66 | 0.04 | 0.02 | 1.9 | 55 | 0.327 | 0.048 |
|  | BED | Healthy | 1.69 | 1.67 | 0.03 | 0.02 | 1.52 | 35 | 0.558 | 0.022 |
|  | BED | OSFED | 1.69 | 1.66 | 0.03 | 0.02 | 1.69 | 50 | 0.447 | 0.033 |
|  | BN | Healthy | 1.66 | 1.67 | -0.01 | 0.01 | -1.04 | 71 | 0.838 | 0.004 |
|  | BN | OSFED | 1.66 | 1.66 | -0.01 | 0.01 | -0.4 | 124 | 0.995 | 0.001 |
|  | Healthy | OSFED | 1.67 | 1.66 | 0.01 | 0.01 | 0.62 | 131 | 0.972 | 0.001 |
| Weight | AN | BED | 51 | 77.11 | -26.11 | 3.08 | -8.48 | 35 | *** | 0.625 |
|  | AN | BN | 51 | 63.98 | -12.98 | 1.82 | -7.12 | 69 | *** | 0.325 |
|  | AN | Healthy | 51 | 65.53 | -14.53 | 0.94 | -15.42 | 526 | *** | 0.205 |
|  | AN | OSFED | 51 | 64.83 | -13.83 | 1.55 | -8.91 | 129 | *** | 0.306 |
|  | BED | BN | 77.11 | 63.98 | 13.13 | 3.47 | 3.79 | 53 | ** | 0.169 |
|  | BED | Healthy | 77.11 | 65.53 | 11.59 | 3.09 | 3.74 | 36 | ** | 0.118 |
|  | BED | OSFED | 77.11 | 64.83 | 12.28 | 3.33 | 3.69 | 47 | ** | 0.148 |
|  | BN | Healthy | 63.98 | 65.53 | -1.55 | 1.85 | -0.84 | 73 | 0.918 | 0.002 |
|  | BN | OSFED | 63.98 | 64.83 | -0.85 | 2.22 | -0.38 | 120 | 0.995 | 0.001 |
|  | Healthy | OSFED | 65.53 | 64.83 | 0.7 | 1.58 | 0.44 | 141 | 0.992 | 0.001 |
| BMI | AN | BED | 18.56 | 27.03 | -8.47 | 1 | -8.46 | 35 | *** | 0.611 |
|  | AN | BN | 18.56 | 23.4 | -4.84 | 0.66 | -7.36 | 68 | *** | 0.35 |
|  | AN | Healthy | 18.56 | 23.51 | -4.95 | 0.31 | -16.12 | 475 | *** | 0.234 |
|  | AN | OSFED | 18.56 | 23.54 | -4.98 | 0.55 | -8.98 | 126 | *** | 0.313 |
|  | BED | BN | 27.03 | 23.4 | 3.63 | 1.16 | 3.13 | 58 | * | 0.116 |
|  | BED | Healthy | 27.03 | 23.51 | 3.52 | 1 | 3.52 | 35 | * | 0.113 |
|  | BED | OSFED | 27.03 | 23.54 | 3.49 | 1.1 | 3.16 | 51 | * | 0.104 |
|  | BN | Healthy | 23.4 | 23.51 | -0.11 | 0.66 | -0.16 | 68 | 1 | 0 |
|  | BN | OSFED | 23.4 | 23.54 | -0.14 | 0.8 | -0.17 | 120 | 1 | 0 |
|  | Healthy | OSFED | 23.51 | 23.54 | -0.03 | 0.56 | -0.06 | 128 | 1 | 0 |

Abbreviations: AN; Anorexia Nervosa, BMI; body mass index, BN; Bulimia Nervosa, BED; Binge Eating Disorder, DF; degrees of freedom, OSFED; Other Specified Feeding or Eating Disorder, SE; standard error.

1. **Correlations**

Table F1. Pearson correlation coefficients across regression variables. Note: Asterisks indicate p-value significance; * < 0.05, ** < 0.01, *** < 0.001.

| Variables | | AN | BN | BED | OSFED | Healthy |
| --- | --- | --- | --- | --- | --- | --- |
| Age | BMI | -0.0138 | 0.2836 | -0.0583 | 0.3047 | 0.4122*** |
|  | EmReg_CR | 0.1332 | 0.0683 | 0.3003 | 0.0796 | 0.1852** |
|  | EmReg_ES | 0.0789 | -0.1154 | 0.2129 | 0.0815 | -0.2203*** |
|  | CFI_Alt | -0.1363 | 0.2049 | 0.2838 | 0.1747 | 0.0756 |
|  | CFI_Ctrl | 0.1297 | 0.3802 | 0.0913 | -0.0019 | 0.351*** |
|  | YPSQ_EFSA | 0.0764 | 0.3154 | 0.2922 | -0.0318 | 0.3222*** |
|  | YPSQ_S | 0.0434 | 0.1593 | 0.476 | -0.0158 | 0.1407 |
|  | YPSQ_EC | -0.0642 | -0.0014 | 0.3302 | 0.0517 | 0.1011 |
|  | YPSQ_O | 0.0485 | 0.3027 | 0.2225 | -0.0736 | 0.3335*** |
|  | YPSQ_EOS | -0.0442 | 0.2516 | 0.1956 | 0.0031 | 0.2759*** |
|  | YPSQ_SCRE | 0.0693 | 0.2995 | 0.4906 | -0.0739 | 0.3901*** |
|  | YPSQ_DS | 0.1284 | 0.4404 | 0.309 | 0.281 | 0.3746*** |
|  | YPSQ_SB | -0.062 | 0.1983 | 0.2777 | -0.1497 | 0.2723*** |
|  | YPSQ_HSC | 0.051 | 0.1726 | 0.3427 | 0.1259 | 0.2209*** |
|  | EDE_Q_R | 0.1303 | -0.4085 | -0.455 | 0.0052 | -0.2264*** |
|  | EDE_Q_W | 0.0611 | -0.0437 | -0.4293 | 0.137 | -0.2863*** |
|  | EDE_Q_S | 0.0798 | -0.1416 | -0.404 | 0.1126 | -0.2895*** |
|  | EDE_Q_E | -0.0406 | -0.3741 | -0.4371 | -0.0556 | -0.3802*** |
|  | EDE_Q_G | 0.0712 | -0.2817 | -0.4709 | 0.0655 | -0.318*** |
| BMI | EmReg_CR | 0.0611 | -0.2275 | -0.1401 | -0.0218 | 0.0387 |
|  | EmReg_ES | -0.004 | -0.1721 | 0.2703 | 0.0633 | -0.0851 |
|  | CFI_Alt | 0.0367 | 0.0815 | 0.2036 | 0.0169 | 0.0363 |
|  | CFI_Ctrl | 0.194 | 0.0266 | -0.0792 | -0.0969 | 0.1993** |
|  | YPSQ_EFSA | 0.0372 | -0.109 | -0.444 | 0.0683 | 0.106 |
|  | YPSQ_S | 0.2802 | 0.0305 | -0.2118 | 0.0624 | 0.0946 |
|  | YPSQ_EC | 0.0106 | 0.0644 | -0.0713 | -0.091 | 0.0053 |
|  | YPSQ_O | 0.1116 | -0.1733 | -0.597* | -0.0044 | 0.1127 |
|  | YPSQ_EOS | -0.0092 | -0.0103 | -0.3719 | -0.0453 | 0.1175 |
|  | YPSQ_SCRE | 0.1638 | 0.0421 | -0.5383 | -0.1655 | 0.1718* |
|  | YPSQ_DS | 0.1067 | 0.1548 | -0.0545 | 0.0409 | 0.1516 |
|  | YPSQ_SB | 0.1864 | 0.1121 | -0.5994* | -0.0886 | 0.1589 |
|  | YPSQ_HSC | 0.1913 | -0.1099 | -0.4318 | -0.1106 | -0.0416 |
|  | EDE_Q_R | -0.2242 | 0.0083 | 0.2428 | 0.0539 | -0.0335 |
|  | EDE_Q_W | -0.1423 | 0.2599 | 0.6028* | 0.2802 | 0.1007 |
|  | EDE_Q_S | -0.1597 | 0.156 | 0.6972** | 0.2481 | 0.0789 |
|  | EDE_Q_E | -0.2697 | -0.0437 | 0.4698 | 0.0812 | -0.0402 |
|  | EDE_Q_G | -0.2222 | 0.1109 | 0.5921 | 0.1943 | 0.0356 |
| EmReg_CR | EmReg_ES | 0.0752 | -0.0999 | 0.2419 | -0.1377 | -0.2167*** |
|  | CFI_Alt | 0.4113*** | 0.2277 | 0.3022 | 0.3826* | 0.385*** |
|  | CFI_Ctrl | 0.4174*** | 0.3969 | -0.0578 | 0.411** | 0.4718*** |
|  | YPSQ_EFSA | 0.3747*** | 0.3573 | 0.4458 | 0.4779*** | 0.4138*** |
|  | YPSQ_S | 0.3508** | 0.4365 | 0.4746 | 0.1346 | 0.3854*** |
|  | YPSQ_EC | 0.1136 | 0.144 | 0.5108 | 0.0253 | 0.1662* |
|  | YPSQ_O | 0.3305** | 0.2985 | 0.6902** | 0.4772*** | 0.5104*** |
|  | YPSQ_EOS | 0.1858 | 0.062 | 0.4537 | 0.3641 | 0.2915*** |
|  | YPSQ_SCRE | 0.3917*** | 0.1817 | 0.6493** | 0.3648 | 0.4865*** |
|  | YPSQ_DS | 0.157 | 0.3498 | 0.5525 | 0.3606 | 0.2834*** |
|  | YPSQ_SB | 0.3179* | 0.1922 | 0.5652 | 0.4951*** | 0.377*** |
|  | YPSQ_HSC | 0.3841*** | 0.2371 | 0.5274 | 0.3155 | 0.3762*** |
|  | EDE_Q_R | -0.192 | -0.1262 | -0.3047 | -0.1277 | -0.306*** |
|  | EDE_Q_W | -0.2033 | -0.3231 | -0.4304 | -0.1456 | -0.3878*** |
|  | EDE_Q_S | -0.188 | -0.2046 | -0.3837 | -0.1903 | -0.3884*** |
|  | EDE_Q_E | -0.1581 | -0.2253 | -0.431 | -0.2629 | -0.3995*** |
|  | EDE_Q_G | -0.2108 | -0.2492 | -0.4287 | -0.1998 | -0.403*** |
| EmReg_ES | CFI_Alt | -0.0065 | 0.0304 | 0.1015 | -0.1217 | -0.1397 |
|  | CFI_Ctrl | -0.1998 | -0.3762 | -0.4692 | -0.3628 | -0.3134*** |
|  | YPSQ_EFSA | -0.2517 | -0.424 | -0.3553 | -0.3345 | -0.442*** |
|  | YPSQ_S | -0.1196 | -0.1912 | -0.3257 | -0.351 | -0.2813*** |
|  | YPSQ_EC | 0.1607 | -0.0021 | 0.3102 | 0.2096 | -0.0732 |
|  | YPSQ_O | -0.1504 | -0.3084 | -0.0921 | -0.3303 | -0.3629*** |
|  | YPSQ_EOS | -0.6374*** | -0.5151* | -0.413 | -0.7108*** | -0.7137*** |
|  | YPSQ_SCRE | -0.161 | -0.4212 | 0.111 | -0.3962* | -0.4373*** |
|  | YPSQ_DS | 0.0035 | -0.3307 | 0.1791 | -0.0401 | -0.2618*** |
|  | YPSQ_SB | -0.2773 | -0.6191*** | -0.1518 | -0.3946* | -0.3877*** |
|  | YPSQ_HSC | 0.0437 | -0.1326 | 0.2396 | -0.126 | -0.243*** |
|  | EDE_Q_R | 0.1155 | 0.2362 | 0.1183 | 0.352 | 0.3247*** |
|  | EDE_Q_W | 0.0828 | 0.2632 | 0.1137 | 0.2551 | 0.344*** |
|  | EDE_Q_S | 0.1375 | 0.3695 | 0.167 | 0.3084 | 0.3815*** |
|  | EDE_Q_E | 0.1997 | 0.3073 | 0.1198 | 0.3344 | 0.4032*** |
|  | EDE_Q_G | 0.1519 | 0.3554 | 0.1487 | 0.3479 | 0.3957*** |
| CFI_Alt | CFI_Ctrl | 0.2907* | 0.0039 | -0.0424 | 0.257 | 0.3271*** |
|  | YPSQ_EFSA | 0.3097* | 0.1345 | 0.2409 | 0.1871 | 0.2274*** |
|  | YPSQ_S | 0.3489** | 0.1384 | 0.396 | 0.2575 | 0.363*** |
|  | YPSQ_EC | 0.2353 | 0.3359 | 0.4189 | 0.2277 | 0.2325*** |
|  | YPSQ_O | 0.1934 | 0.0265 | -0.0012 | 0.1941 | 0.2321*** |
|  | YPSQ_EOS | 0.1707 | 0.2006 | 0.223 | 0.2277 | 0.2359*** |
|  | YPSQ_SCRE | 0.1669 | -0.2409 | 0.0011 | 0.0168 | 0.2474*** |
|  | YPSQ_DS | 0.0544 | 0.1071 | 0.3823 | 0.2647 | 0.2076*** |
|  | YPSQ_SB | 0.2469 | -0.003 | 0.257 | 0.193 | 0.2279*** |
|  | YPSQ_HSC | 0.4262*** | 0.321 | 0.2215 | 0.3665 | 0.2843*** |
|  | EDE_Q_R | -0.084 | 0.2977 | 0.1133 | -0.0094 | -0.0858 |
|  | EDE_Q_W | -0.0551 | 0.2716 | 0.1911 | 0.0903 | -0.137 |
|  | EDE_Q_S | -0.0227 | 0.3085 | 0.2066 | 0.0253 | -0.1299 |
|  | EDE_Q_E | -0.0016 | 0.0653 | 0.1445 | -0.1096 | -0.1421 |
|  | EDE_Q_G | -0.046 | 0.2852 | 0.1885 | 0.0047 | -0.1343 |
| CFI_Ctrl | YPSQ_EFSA | 0.3692*** | 0.5623** | 0.0748 | 0.5087*** | 0.45*** |
|  | YPSQ_S | 0.3684*** | 0.5243** | 0.397 | 0.4954*** | 0.5035*** |
|  | YPSQ_EC | 0.0845 | -0.058 | -0.0352 | -0.0966 | 0.1614 |
|  | YPSQ_O | 0.3881*** | 0.6169*** | 0.1912 | 0.5244*** | 0.5807*** |
|  | YPSQ_EOS | 0.2429 | 0.2968 | 0.1712 | 0.4463** | 0.3485*** |
|  | YPSQ_SCRE | 0.4249*** | 0.5885*** | 0.1059 | 0.4556*** | 0.5719*** |
|  | YPSQ_DS | 0.2687 | 0.4656 | 0.2115 | 0.3637 | 0.4275*** |
|  | YPSQ_SB | 0.3831*** | 0.477* | 0.2993 | 0.5411*** | 0.4751*** |
|  | YPSQ_HSC | 0.4309*** | 0.3859 | 0.1627 | 0.4729*** | 0.4696*** |
|  | EDE_Q_R | -0.1992 | -0.4335 | -0.3131 | -0.1186 | -0.3234*** |
|  | EDE_Q_W | -0.228 | -0.4548 | -0.147 | -0.1017 | -0.4224*** |
|  | EDE_Q_S | -0.2508 | -0.4312 | -0.204 | -0.2051 | -0.4629*** |
|  | EDE_Q_E | -0.3162* | -0.3868 | -0.2204 | -0.2328 | -0.4676*** |
|  | EDE_Q_G | -0.2804 | -0.4991* | -0.2385 | -0.1858 | -0.4588*** |
| YPSQ_EFSA | YPSQ_S | 0.3395** | 0.235 | 0.7772*** | 0.3705* | 0.4251*** |
|  | YPSQ_EC | -0.0427 | 0.0918 | 0.3311 | 0.0152 | 0.2778*** |
|  | YPSQ_O | 0.5412*** | 0.6234*** | 0.7384*** | 0.7142*** | 0.6681*** |
|  | YPSQ_EOS | 0.3574*** | 0.5206** | 0.8401*** | 0.4362** | 0.4758*** |
|  | YPSQ_SCRE | 0.5007*** | 0.4935* | 0.6219* | 0.6441*** | 0.6416*** |
|  | YPSQ_DS | 0.173 | 0.3224 | 0.2424 | 0.2738 | 0.4863*** |
|  | YPSQ_SB | 0.6097*** | 0.6961*** | 0.7218*** | 0.7274*** | 0.6033*** |
|  | YPSQ_HSC | 0.2314 | 0.1893 | 0.4432 | 0.3036 | 0.3752*** |
|  | EDE_Q_R | -0.2049 | -0.4256 | -0.2095 | -0.3218 | -0.4559*** |
|  | EDE_Q_W | -0.3612*** | -0.5661** | -0.4151 | -0.2613 | -0.5304*** |
|  | EDE_Q_S | -0.3215** | -0.4546 | -0.4749 | -0.3063 | -0.5519*** |
|  | EDE_Q_E | -0.3369** | -0.405 | -0.4331 | -0.4056* | -0.5274*** |
|  | EDE_Q_G | -0.3453** | -0.5351** | -0.4411 | -0.3556 | -0.564*** |
| YPSQ_S | YPSQ_EC | -0.0847 | -0.0743 | 0.4113 | -0.0247 | 0.1066 |
|  | YPSQ_O | 0.3243** | 0.5131* | 0.5769 | 0.3653 | 0.4907*** |
|  | YPSQ_EOS | 0.2693 | 0.201 | 0.7619*** | 0.447** | 0.3695*** |
|  | YPSQ_SCRE | 0.312* | 0.476* | 0.5246 | 0.3365 | 0.4139*** |
|  | YPSQ_DS | 0.151 | 0.409 | 0.5119 | 0.1799 | 0.3494*** |
|  | YPSQ_SB | 0.2778 | 0.3282 | 0.6961** | 0.3807* | 0.479*** |
|  | YPSQ_HSC | 0.4839*** | 0.5312** | 0.4157 | 0.4537*** | 0.4692*** |
|  | EDE_Q_R | -0.1998 | -0.152 | -0.3654 | -0.1078 | -0.1783* |
|  | EDE_Q_W | -0.233 | -0.1915 | -0.3761 | -0.1025 | -0.297*** |
|  | EDE_Q_S | -0.24 | -0.1363 | -0.3825 | -0.1675 | -0.3352*** |
|  | EDE_Q_E | -0.2522 | -0.2227 | -0.4566 | -0.1528 | -0.3299*** |
|  | EDE_Q_G | -0.2624 | -0.1996 | -0.4342 | -0.1511 | -0.3143*** |
| YPSQ_EC | YPSQ_O | 0.0278 | 0.0769 | 0.3492 | -0.0171 | 0.2367*** |
|  | YPSQ_EOS | 0.0247 | 0.2324 | 0.2446 | -0.0408 | 0.188** |
|  | YPSQ_SCRE | 0.1017 | 0.0133 | 0.2992 | 0.0826 | 0.2891*** |
|  | YPSQ_DS | 0.2374 | 0.0221 | 0.3987 | 0.2461 | 0.1811** |
|  | YPSQ_SB | 0.0631 | 0.0986 | 0.3659 | -0.1111 | 0.2431*** |
|  | YPSQ_HSC | 0.2681 | -0.0745 | 0.3628 | 0.2042 | 0.1947** |
|  | EDE_Q_R | -0.0648 | 0.0705 | -0.2731 | 0.1484 | -0.1456 |
|  | EDE_Q_W | 0.1048 | 0.0207 | -0.1974 | 0.1385 | -0.1993** |
|  | EDE_Q_S | 0.0364 | -0.0106 | -0.1363 | 0.1185 | -0.1853** |
|  | EDE_Q_E | 0.0503 | -0.1157 | -0.3004 | 0.194 | -0.1756* |
|  | EDE_Q_G | 0.0323 | -0.0107 | -0.235 | 0.161 | -0.1922** |
| YPSQ_O | YPSQ_EOS | 0.2586 | 0.3147 | 0.6324* | 0.465*** | 0.4277*** |
|  | YPSQ_SCRE | 0.588*** | 0.6589*** | 0.8131*** | 0.6047*** | 0.7013*** |
|  | YPSQ_DS | 0.2396 | 0.3798 | 0.387 | 0.3372 | 0.4794*** |
|  | YPSQ_SB | 0.3015* | 0.5247** | 0.7333*** | 0.6726*** | 0.5616*** |
|  | YPSQ_HSC | 0.2199 | 0.3746 | 0.6723** | 0.3752* | 0.381*** |
|  | EDE_Q_R | -0.3* | -0.5443** | -0.2804 | -0.2064 | -0.4184*** |
|  | EDE_Q_W | -0.3832*** | -0.4891* | -0.532 | -0.282 | -0.5304*** |
|  | EDE_Q_S | -0.3965*** | -0.4857* | -0.5796 | -0.3795* | -0.5491*** |
|  | EDE_Q_E | -0.3998*** | -0.3704 | -0.4919 | -0.3194 | -0.5401*** |
|  | EDE_Q_G | -0.4203*** | -0.5554** | -0.5412 | -0.3383 | -0.5564*** |
| YPSQ_EOS | YPSQ_SCRE | 0.3226** | 0.3776 | 0.5491 | 0.4571*** | 0.5066*** |
|  | YPSQ_DS | 0.1895 | 0.0455 | 0.3732 | 0.1634 | 0.3138*** |
|  | YPSQ_SB | 0.4676*** | 0.4857* | 0.694** | 0.5615*** | 0.4999*** |
|  | YPSQ_HSC | 0.134 | 0.145 | 0.3947 | 0.2425 | 0.2907*** |
|  | EDE_Q_R | -0.082 | -0.2562 | -0.096 | -0.2031 | -0.2951*** |
|  | EDE_Q_W | -0.0602 | -0.3946 | -0.3104 | -0.1784 | -0.3455*** |
|  | EDE_Q_S | -0.1465 | -0.3866 | -0.4163 | -0.2005 | -0.362*** |
|  | EDE_Q_E | -0.1645 | -0.4721 | -0.3525 | -0.2012 | -0.3631*** |
|  | EDE_Q_G | -0.1312 | -0.4403 | -0.3479 | -0.2188 | -0.3724*** |
| YPSQ_SCRE | YPSQ_DS | 0.248 | 0.2623 | 0.4453 | 0.1279 | 0.4268*** |
|  | YPSQ_SB | 0.3624*** | 0.5243** | 0.7214*** | 0.5953*** | 0.5467*** |
|  | YPSQ_HSC | 0.165 | 0.2955 | 0.6361* | 0.2369 | 0.3922*** |
|  | EDE_Q_R | -0.3842*** | -0.6321*** | -0.3325 | -0.3637 | -0.4969*** |
|  | EDE_Q_W | -0.4523*** | -0.6362*** | -0.5865 | -0.3763* | -0.5812*** |
|  | EDE_Q_S | -0.4869*** | -0.7062*** | -0.6229* | -0.4613*** | -0.6048*** |
|  | EDE_Q_E | -0.4918*** | -0.6304*** | -0.5725 | -0.3685 | -0.604*** |
|  | EDE_Q_G | -0.5164*** | -0.7703*** | -0.6029* | -0.4458** | -0.6232*** |
| YPSQ_DS | YPSQ_SB | 0.1793 | 0.286 | 0.4238 | 0.1098 | 0.4119*** |
|  | YPSQ_HSC | 0.2163 | 0.3801 | 0.5583 | 0.3467 | 0.2476*** |
|  | EDE_Q_R | -0.112 | -0.1531 | -0.1052 | 0.1466 | -0.2281*** |
|  | EDE_Q_W | -0.1188 | -0.1233 | -0.1963 | 0.1544 | -0.2668*** |
|  | EDE_Q_S | -0.1642 | -0.1072 | -0.2586 | 0.1288 | -0.3023*** |
|  | EDE_Q_E | -0.1686 | -0.2053 | -0.4064 | 0.0269 | -0.3116*** |
|  | EDE_Q_G | -0.1613 | -0.1689 | -0.2722 | 0.1317 | -0.3033*** |
| YPSQ_SB | YPSQ_HSC | 0.2243 | 0.1951 | 0.7096*** | 0.3617 | 0.3418*** |
|  | EDE_Q_R | -0.2509 | -0.2766 | -0.2814 | -0.3143 | -0.2767*** |
|  | EDE_Q_W | -0.2634 | -0.3514 | -0.5407 | -0.264 | -0.3774*** |
|  | EDE_Q_S | -0.2539 | -0.3417 | -0.5834 | -0.2901 | -0.4148*** |
|  | EDE_Q_E | -0.2838 | -0.3762 | -0.4646 | -0.3543 | -0.3943*** |
|  | EDE_Q_G | -0.297* | -0.3928 | -0.5388 | -0.3368 | -0.4022*** |
| YPSQ_HSC | EDE_Q_R | -0.093 | 0.0098 | -0.1609 | 0.0667 | -0.1694* |
|  | EDE_Q_W | 0.0128 | -0.15 | -0.315 | 0.0248 | -0.3528*** |
|  | EDE_Q_S | 0.0045 | -0.1054 | -0.4132 | -0.0213 | -0.3703*** |
|  | EDE_Q_E | -0.0693 | -0.2495 | -0.3172 | -0.0513 | -0.3451*** |
|  | EDE_Q_G | -0.0382 | -0.1393 | -0.3538 | 0.005 | -0.3418*** |
| EDE_Q_R | EDE_Q_W | 0.6747*** | 0.5324** | 0.6043* | 0.6903*** | 0.7091*** |
|  | EDE_Q_S | 0.6417*** | 0.5336** | 0.597* | 0.7205*** | 0.6983*** |
|  | EDE_Q_E | 0.622*** | 0.397 | 0.575 | 0.6483*** | 0.7186*** |
|  | EDE_Q_G | 0.8335*** | 0.7192*** | 0.7421*** | 0.8546*** | 0.8389*** |
| EDE_Q_W | EDE_Q_S | 0.8657*** | 0.8759*** | 0.9389*** | 0.9195*** | 0.939*** |
|  | EDE_Q_E | 0.6342*** | 0.6668*** | 0.8321*** | 0.6536*** | 0.8202*** |
|  | EDE_Q_G | 0.9043*** | 0.9004*** | 0.9516*** | 0.9211*** | 0.9481*** |
| EDE_Q_S | EDE_Q_E | 0.7174*** | 0.7424*** | 0.8607*** | 0.7317*** | 0.8606*** |
|  | EDE_Q_G | 0.9319*** | 0.9435*** | 0.9662*** | 0.9577*** | 0.9634*** |
| EDE_Q_E | EDE_Q_G | 0.8364*** | 0.8238*** | 0.9132*** | 0.8301*** | 0.9198*** |

Abbreviations: Alt; alternate, AN; Anorexia Nervosa, BED; Binge Eating Disorder, BMI; body mass index, BN; Bulimia Nervosa, CR; cognitive reappraisal, Ctrl; control, DF; degrees of freedom, DS; developed self, EC; empathic consideration, EDE_Q_E; eating disorder questionnaire eating concern, EDE_Q_G; eating disorder questionnaire global score, EDE_Q_R; eating disorder questionnaire restraint, EDE_Q_S; eating disorder questionnaire shape concern, EDE_Q_W; eating disorder questionnaire weight concern, EFSA; emotional fulfilment and stable attachment, EOS; emotional openness, ES; expressive suppression, HSC; healthy self-control, O; openness, OSFED; Other Specified Feeding or Eating Disorder, S; success, SB; social belonging, SCRE; self-compassion and realistic expectations.

1. **Power analysis**

The actual statistical power was estimated for each of five final models as a “whole” based on R2 and for each significant “local” effect within each final model based on variance explained by the local effect and residual variance of the model. The main purpose of the power analysis was to examine whether the actual sample size provided statistical power above the thresholds 0.8 which is commonly used in the research (Hanley & Moodie, 2011). Cohen’s f2 was used to determine the effect size measure in post-hoc power analysis for the linear regression for the whole model, as well as local ones: the effects of predictors within each regression (Selya et al., 2012).

The effect size for the whole regression was in range 0.34 ≤ *f*^2^ ≤ 2.08 with minimal value *f*^2^ = 0.34 for model with OSFED as the outcome (*n* = 93, *R*^2^ = 0.25) and maximal value *f*^2^ = 2.08 for model with BN as the outcome (*n* = 55, *R*^2^ = 0.68; See Table G1). Thus, for all models the effect size for the whole effect was close to or exceeded 0.35 which be regarded as a “large” effect size according to Cohen’s guidelines (Cohen, 1988). Power analysis was also conducted with actual sample sizes, which yielded statistical powers that exceeded 0.8 for the whole effect for all five models with *α* = 0.05 (see Table G1, Supplementary Materials). Post hoc statistical power for local effects also exceeded the thresholds 0.8 for all models excluding the model with OSFED as the outcome (see Table G2) with 0.78 for effect of Expressive Suppression, 0.74 for effect of BMI, and 0.72 for effect of Young Positive Schema Questionnaire. Barring OSFED, the sample sizes yielded actual statistical powers above 0.8 at significance level 0.05 with large effect sizes for all other models.

Table G1. Post hoc statistical power for the whole effect of the final models

| Model | N | R^2^ | Effect size f^2^ | Statistical power at Sign. Level α=0.05 |
| --- | --- | --- | --- | --- |
| AN | 155 | 0.30 | 0.44 | >0.99 |
| BN | 55 | 0.68 | 2.08 | >0.99 |
| BED | 33 | 0.64 | 1.76 | 0.96 |
| OSFED | 93 | 0.25 | 0.34 | 0.93 |
| Healthy | 505 | 0.50 | 0.99 | >0.99 |

Table G2**.** Post hoc statistical power for the local effects

| Dependent variable | Predictor | Unique (Semi-Partial-Squared) Variance (%) | Residual variance of the model | N | Effect size f^2^ | Statistical power at α=0.05 |
| --- | --- | --- | --- | --- | --- | --- |
| AN | YPSQ_SCRE | 9.49 | 0.70 | 155 | 0.14 | >0.99 |
|  |  |  |  |  |  |  |
| BN | YPSQ_S | 9.81 | 0.33 | 55 | 0.30 | 0.98 |
| BN | Cognitive Reappraisal | 10.28 | 0.33 | 55 | 0.32 | 0.98 |
| BN | YPSQ_EOS | 10.07 | 0.33 | 55 | 0.31 | 0.98 |
| BN | YPSQ_SCRE | 32.96 | 0.33 | 55 | 1.01 | >0.99 |
| BED | Age | 40.65 | 0.36 | 33 | 1.12 | >0.99 |
| BED | BMI | 32.83 | 0.36 | 33 | 0.91 | >0.99 |
| BED | Cognitive Reappraisal | 26.40 | 0.36 | 33 | 0.73 | >0.99 |
| BED | Alternate | 28.48 | 0.36 | 33 | 0.79 | >0.99 |
| OSFED | BMI | 5.57 | 0.75 | 93 | 0.07 | 0.74 |
| OSFED | Expressive Suppression | 6.17 | 0.75 | 93 | 0.08 | 0.78 |
| OSFED | YPSQ_DS | 5.36 | 0.75 | 93 | 0.07 | 0.72 |
| Healthy | Age | 2.37 | 0.50 | 505 | 0.05 | >0.99 |
| Healthy | BMI | 6.04 | 0.50 | 505 | 0.12 | >0.99 |
| Healthy | Sex | 1.53 | 0.50 | 505 | 0.03 | 0.98 |
| Healthy | Cognitive Reappraisal | 0.95 | 0.50 | 505 | 0.02 | 0.87 |
| Healthy | Expressive Suppression | 2.09 | 0.50 | 505 | 0.04 | 1.00 |
| Healthy | Alternate | 0.92 | 0.50 | 505 | 0.02 | 0.86 |
| Healthy | Control | 0.96 | 0.50 | 505 | 0.02 | 0.87 |
| Healthy | YPSQ_EFSA | 3.64 | 0.50 | 505 | 0.07 | >0.99 |
| Healthy | YPSQ_SCRE | 7.50 | 0.50 | 505 | 0.15 | >0.99 |

**References**

Hanley, J., & Moodie, E. (2011). Sample Size, Precision and Power Calculations: A Unifed Approach. *Journal of Biometrics & Biostatistics*, *2*(5), 1-9. <https://doi.org/10.4172/2155-6180.1000124>

Selya, A. S., Rose Js Fau - Dierker, L. C., Dierker Lc Fau - Hedeker, D., Hedeker D Fau - Mermelstein, R. J., & Mermelstein, R. J. (2012). A Practical Guide to Calculating Cohen's f(2), a Measure of Local Effect Size, from PROC MIXED. (1664-1078 (Electronic)).
