# Supplementary material for: Early adaptive schemas, emotional regulation, and cognitive flexibility in eating disorders: subtype specific predictors of eating disorder symptoms using hierarchical linear regression
Source: Eat Weight Disord. 2024 Aug 29;29(1):54. doi: 10.1007/s40519-024-01682-4 (PMC11362190; doi:10.1007/s40519-024-01682-4)
Supplement: Supplementary file 2 — Supplementary material 2. [file 40519_2024_1682_MOESM2_ESM.pdf]

Early adaptive schemas, emotional regulation, and cognitive flexibility in eating disorders: sub-type specific predictors of eating disorder symptoms using hierarchical linear regression

Introduction

Here we detail the manuscripts analysis of the Anorexia Nervosa sample using the Hierarchical Linear Regression (HLR: version v0.2.2) package. We cover the:

- Analysis process
- Outputs (assumption checks and model statistics)
- Validation of outputs gainst the equivalent analysis performed in SPSS.

Setup

Import the necessary packages, set directory, and load data file.

- Check HLR dependencies at https://github.com/teanijarv/HLR

```
import os
import statsmodels as sm
import pandas as pd
import numpy as np
from HLR import HierarchicalLinearRegression

# Set working directory as required
os.chdir('c:\\')

# Load datasets
data = pd.read_csv('AnorexiaDataset_Final.csv')
```

Setup

```
# Define the models for hierarchical regression including predictors for each model
X = {1: ['Age', 'Sex', 'BMI'],
     2: ['Age', 'Sex', 'BMI', 'EmReg_CR', 'EmReg_ES','CFI_Alt', 'CFI_Ctrl'],
     3: ['Age', 'Sex', 'BMI','EmReg_CR', 'EmReg_ES','CFI_Alt', 'CFI_Ctrl','YPSQ_EFSA',
        'YPSQ_S', 'YPSQ_EC', 'YPSQ_O','YPSQ_EOS', 'YPSQ_SCRE', 'YPSQ_DS', 'YPSQ_SB', 'YPSQ_HSC']}

# Define the outcome variable
y = 'EDE_Q_G'

# Initiate the HLR object (missing_data and ols_params are optional parameters)
hreg = HierarchicalLinearRegression(data, X, y)
```

Output Summary

Generate a summarised report of HLR and output (if desired)

```
hreg.summary()
summary = hreg.summary()
summary.to_csv('model_summary.csv')
```

Model Level	Predictors	N (observations)	DF (residuals)	DF (model)	R-squared	F-value	P-value (F)	SSR	SSTO	...	MSE (total)	Beta coefs
1	[Age, Sex, BMI]	155.0	151.0	3.0	0.056637	3.021874	3.159795e-02	200.285981	212.310597	...	1.37864	{'const': 5.28540529816[illegible] 'Age': 0.011450[illegible]
2	[Age, Sex, BMI, EmReg_CR, EmReg_ES, CFI_Alt, C...	155.0	147.0	7.0	0.155811	3.875933	6.674191e-04	179.230364	212.310597	...	1.37864	{'const': 4.97434661265[illegible] 'Age': 0.020813[illegible]

Model Level	Predictors	N (observations)	DF (residuals)	DF (model)	R-squared	F-value	P-value (F)	SSR	SSTO	...	MSE (total)	Beta coefs
3	[Age, Sex, BMI, EmReg_CR, EmReg_ES, CFI_Alt, C...	155.0	138.0	16.0	0.375967	5.196387	2.048347e-08	132.488790	212.310597	...	1.37864	{'const': 4.412113500863 'Age': 0.0212693

Assumption Checks

Run diagnostics on all the models. Convert dictionary output to dataframe and output

```
diagnostics = hreg.diagnostics(verbose=True)
diagnostics = pd.DataFrame.from_dict(diagnostics)
diagnostics.to_csv('model_diagnostics.csv')
```

Test	Result	Statistic	p-value	Passed
Independence of Residuals (Durbin-Watson)	False	1.1884652006141259	-	No
Linearity (Pearson r)				
- Age	False	0.07116642137977928	0.3788813749802866	No
- Sex	False	0.044998147255722064	0.5782317894531495	No
- BMI	True	-0.22216787322709358	0.005462924797018646	Yes
- EmReg_CR	True	-0.21084450482551603	0.008453050971642742	Yes
- EmReg_ES	False	0.1519284443242815	0.059139738484216386	No
- CFI_Alt	False	-0.046043777241197489	0.5694225757304743	No
- CFI_Ctrl	True	-0.2804165610693205	0.00040920428876663493	Yes
- YPSQ_EFSA	True	-0.34527010801913754	1.0832045204506238e-05	Yes
- YPSQ_S	True	-0.26235550388827794	0.000974376710118128	Yes
- YPSQ_EC	False	0.032340878753329010	0.689535128316351	No
- YPSQ_O	True	-0.4203043607770755	5.189116219910229e-08	Yes
- YPSQ_EOS	False	-0.13122824398530147	0.10360969550479024	No
- YPSQ_SCRE	True	-0.5164286115339844	6.061744997756237e-12	Yes
- YPSQ_DS	True	-0.1612898480338923	0.04497001142293863	Yes
- YPSQ_SB	True	-0.29695298580374724	0.00017535580981943336	Yes
- YPSQ_HSC	False	-0.03817329213893438	0.6372275950047485	No
Linearity (Rainbow Test)	False	2.7297286583564198	3.9498634690229417e-05	No
Homoscedasticity (Breusch-Pagan Test)	True	16.78137858185311	0.39987855337467015	Yes
Homoscedasticity (Goldfeld-Quandt Test)	True	0.4497303098697752	0.9988608971662183	Yes
Multicollinearity (Pairwise Correlations)	True			Yes
Multicollinearity (VIFs)	True			Yes
Outliers (Extreme Standardized Residuals)	True			Yes
Outliers (High Cook's Distance)	True			Yes
Normality (Mean of Residuals)	True	1.5815306054015132e-14	-	Yes
Normality (Shapiro-Wilk Test)	False	0.9612044095993042	0.00024456685059703887	No

Plots

Generate plots to verify model assumptions.

```
hreg.plot_studentized_residuals_vs_fitted()
hreg.plot_qq_residuals()
hreg.plot_influence()
hreg.plot_std_residuals()
hreg.plot_histogram_std_residuals()
```

```
hreg.plot_partial_regression()
```

Here we show the model 3 studentized_residuals_vs_fitted plot.

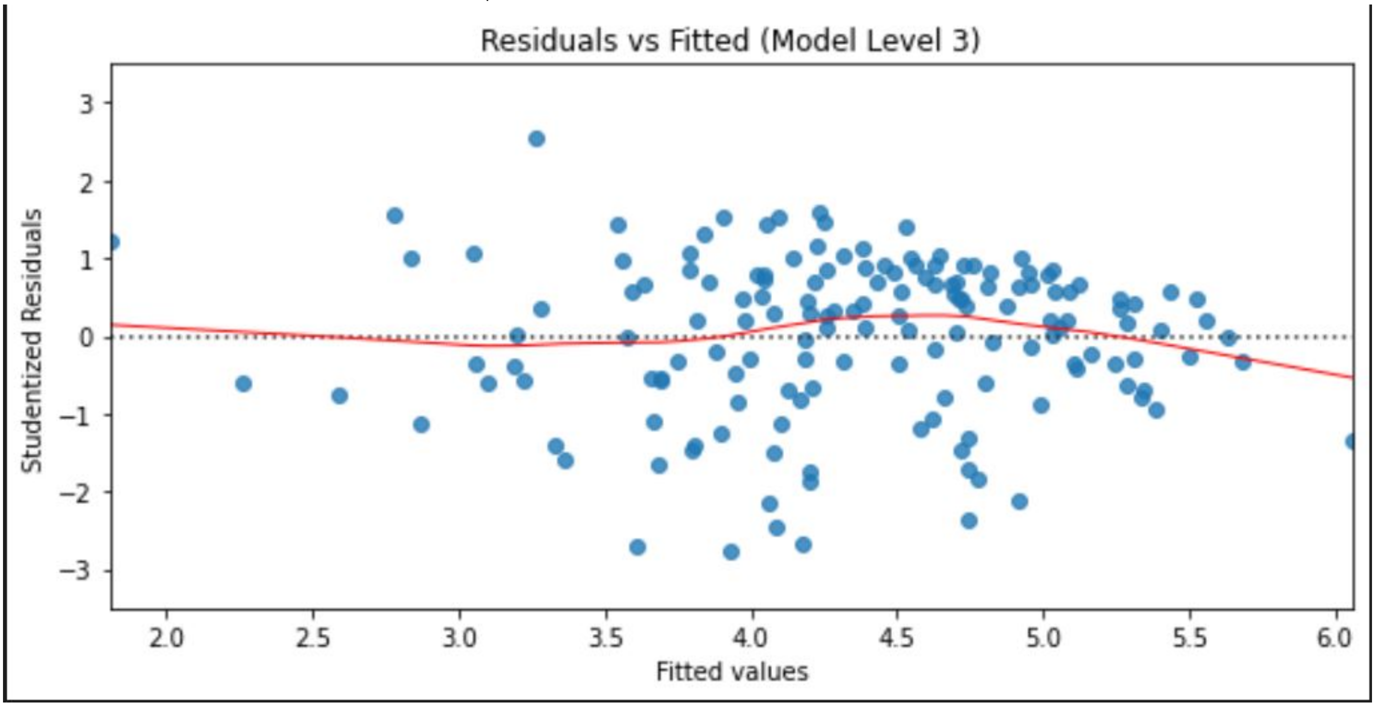

SPSS Validation

The above HLR results are compared to equivalent analyses conducted with SPSS (v29.0).

Model summary results (including Durbin-Watson).

SPSS

Model Summary[d]

Model	R	R Square	Adjusted R Square	Std. Error of the Estimate	Change Statistics		Durbin-Watson
					R Square Change	F Change	
1	.238[a]	.057	.038	1.15169	.057	3.022	
2	.395[b]	.156	.116	1.10420	.099	4.317	
3	.613[c]	.376	.304	.97983	.220	5.410	1.188

HLR

Model Level	R-squared	R-squared change	F-value change	P-value (F-value change)
0	0.056637	NaN	NaN	NaN
1	0.155811	0.099174	4.317315	0.002485
2	0.375967	0.220157	5.409545	0.000002

Test	Result	Statistic	p-value	Passed
Independence of Residuals (Durbin-Watson)	False	1.1884652006141259	-	No

ANOVA results

SPSS

ANOVA[a]

Model		Sum of Squares	df	Mean Square	F	Sig.
1	Regression	12.025	3	4.008	3.022	.032[b]
	Residual	200.286	151	1.326		
	Total	212.311	154			
2	Regression	33.080	7	4.726	3.876	<.001[c]
	Residual	179.230	147	1.219		
	Total	212.311	154			
3	Regression	79.822	16	4.989	5.196	<.001[d]
	Residual	132.489	138	.960		
	Total	212.311	154			

HLR

Model Level	N (observations)	DF (residuals)	DF (model)	R-squared	F-value	P-value (F)	SSR	SSTO	MSE (model)	MSE (residuals)	MSE (total)
0	1	155.0	151.0	3.0	0.056637	3.021874e-02	200.285981	212.310597	4.008205	1.326397	1.37864
1	2	155.0	147.0	7.0	0.155811	6.674191e-04	179.230364	212.310597	4.725748	1.219254	1.37864
2	3	155.0	138.0	16.0	0.375967	2.048347e-08	132.488790	212.310597	4.988863	0.960064	1.37864

Predictor	VIF
Age	1.14286
Sex	1.11053
BMI	1.20749
EmReg_CR	1.67596
EmReg_ES	2.05902
CFI_Alt	1.59785
CFI_Ctrl	1.76409
YPSQ_EFSA	2.43041
YPSQ_S	1.76715
YPSQ_EC	1.37120
YPSQ_O	1.85001
YPSQ_EOS	2.31678
YPSQ_SCRE	1.93756
YPSQ_DS	1.28125
YPSQ_SB	2.06255
YPSQ_HSC	1.83167

Beta coefficients, significance, and partial and semi-partial (part) correlation, and VIF results.

SPSS

Coefficients[a]

Model		Unstandardized Coefficients		Standardized Coefficients			Correlations			Collinearity Statistics	
		B	Std. Error	Beta	t	Sig.	Zero-order	Partial	Part	Tolerance	VIF
3	(Constant)	4.412	1.207		3.656	<.001					
	Age	.021	.012	.128	1.780	.077	.071	.150	.120	.875	1.143
	Sex	.424	.399	.075	1.062	.290	.045	.090	.071	.900	1.111
	BMI	-.042	.032	-.097	-1.316	.190	-.222	-.111	-.088	.828	1.207
	EmReg_CR	-.055	.088	-.054	-.621	.536	-.211	-.053	-.042	.597	1.676
	EmReg_ES	.104	.087	.116	1.203	.231	.152	.102	.081	.486	2.059
	CFI_Alt	.008	.008	.076	.890	.375	-.046	.076	.060	.626	1.598
	CFI_Ctrl	-.004	.013	-.029	-.325	.745	-.280	-.028	-.022	.567	1.764
	YPSQ_EFSA	-.022	.094	-.025	-.237	.813	-.345	-.020	-.016	.411	2.430
	YPSQ_S	-.143	.088	-.144	-1.616	.108	-.262	-.136	-.109	.566	1.767
	YPSQ_EC	.030	.094	.025	.320	.749	.032	.027	.022	.729	1.371
	YPSQ_O	-.158	.095	-.151	-1.656	.100	-.420	-.140	-.111	.541	1.850
	YPSQ_EOS	.196	.101	.199	1.947	.054	-.131	.163	.131	.432	2.317
	YPSQ_SCRE	-.469	.123	-.356	-3.804	<.001	-.516	-.308	-.256	.516	1.938
	YPSQ_DS	-.058	.064	-.070	-.916	.361	-.161	-.078	-.062	.780	1.281
	YPSQ_SB	-.112	.095	-.114	-1.182	.239	-.297	-.100	-.080	.485	2.063
	YPSQ_HSC	.131	.080	.149	1.639	.103	-.038	.138	.110	.546	1.832

HLR (Note: Output formatted to allow for ease of comparison)

	Beta coefs	Std Beta coefs	P-values (beta coefs)	Partial correlations	Semi-partial (part) correlations
Constant	4.412114		0.000363		
'Age'	0.021269	0.127973	0.077252	0.149826	0.119707
'Sex'	0.424315	0.075286	0.289912	0.09007	0.071442
'BMI'	-0.04237	-0.09723	0.190423	-0.11131	-0.08848
'EmReg_CR'	-0.05474	-0.05402	0.535923	-0.05275	-0.04173
'EmReg_ES'	0.104368	0.116073	0.231067	0.101867	0.080891
'CFI_Alt'	0.007554	0.075682	0.37483	0.075575	0.059872
'CFI_Ctrl'	-0.00407	-0.02906	0.745369	-0.02769	-0.02188
'YPSQ_EFSA'	-0.02224	-0.02488	0.812764	-0.0202	-0.01596
'YPSQ_S'	-0.14268	-0.14442	0.10846	-0.13625	-0.10864
'YPSQ_EC'	0.030073	0.025203	0.749401	0.027236	0.021523
'YPSQ_O'	-0.15803	-0.15148	0.09995	-0.13961	-0.11137
'YPSQ_EOS'	0.196106	0.19927	0.053582	0.163498	0.130918
'YPSQ_SCRE'	-0.46869	-0.3561	0.000213	-0.30809	-0.25583
'YPSQ_DS'	-0.05848	-0.06976	0.361025	-0.07778	-0.06163
'YPSQ_SB'	-0.11196	-0.11419	0.239075	-0.10015	-0.07951
'YPSQ_HSC'	0.13052	0.14921	0.10339	0.13822	0.11025

Using HLR

HLR is a simple Python package for running hierarchical linear regression.

It is built to work with Pandas dataframes, uses SciPy, statsmodels, and pingouin under the hood, and runs diagnostic tests for testing assumptions while plotting figures with matplotlib and seaborn.

Check out and contribute to the code at https://github.com/teanijarv/HLR.

References

Anijärv, T. E., Mitchell, J. and Boyle, R. (2024) 'teanijarv/HLR: v0.2.2'. Zenodo. https://doi.org/10.5281/zenodo.7683808

Mitchell, J. S., Huckstepp, T., Allen, A., Louis, P. J., Anijärv, T. E., & Hermens, D. F. (2024:Under review). Early adaptive schemas, emotional regulation, and cognitive flexibility in eating disorders: An examination of sub-type specific predictors of eating disorder symptoms using hierarchical linear regression
